# Supplementary material for: An in vitro model of tumor heterogeneity resolves genetic, epigenetic, and stochastic sources of cell state variability
Source: PLoS Biol. 2021 Jun 1;19(6):e3000797. doi: 10.1371/journal.pbio.3000797 (PMC8195356; doi:10.1371/journal.pbio.3000797)
Supplement: S1 Text — Four subsections are included, discussing (i) descriptions of the genetic, epigenetic, and stochastic levels of heterogeneity believed to coexist within tumors, (ii) the fundamental relationship between transcriptomics and epigenetics, (iii) the rationale behind the genetic-to-epigenetic correlation metric utilized in this work, and (iv) 2 hypotheses regarding the origins of the DS8 genetic mutant subline. Three tables are also included containing (A) a glossary of terms, (B) a list of genes associated with mutation heatmaps in Figs 3D and 4D, and (C) rate parameters used for stochastic simulations. (PDF) [file pbio.3000797.s001.pdf]

SUPPLEMENTARY TEXT FOR

An *in vitro* model of tumor heterogeneity resolves genetic, epigenetic, and stochastic sources of cell state variability

Corey E. Hayford[1], Darren R. Tyson[2], C. Jack Robbins III[2†], Peter L. Frick[1#], Vito Quaranta[2,3], and Leonard A. Harris[4,5,6*]

[1]Chemical and Physical Biology Graduate Program, [2]Department of Biochemistry, and [3]Department of Pharmacology, Vanderbilt University School of Medicine, Nashville, TN USA

[4]Department of Biomedical Engineering and [5]Interdisciplinary Graduate Program in Cell and Molecular Biology, University of Arkansas, Fayetteville, AR USA

[6]Cancer Biology Program, Winthrop P. Rockefeller Cancer Institute, University of Arkansas for Medical Sciences, Little Rock, AR USA

[†]Current address: MD-PhD Graduate Program, Yale University School of Medicine, New Haven, CT USA
[#]Current address: Intuit Inc., Mountain View, CA USA

***Address correspondence to:** Leonard A. Harris, harrisl@uark.edu

Table of Contents

Genetic, epigenetic, and stochastic sources of tumor heterogeneity 1
Relationship between transcriptomics and epigenetics 2
The genetic-to-epigenetic connection 3
Multiple possible routes to a new genetic state in DS8 4
Table A. Glossary of relevant terms. 6
Table B. List of genes associated with mutation heatmaps in Figs 3D and 4D. 8
Table C. Rate parameters used for monoclonal and polyclonal growth models. 9
Supplementary References 10

Genetic, epigenetic, and stochastic sources of tumor heterogeneity

Based on prior work in this area[1–7], we view intratumoral heterogeneity as comprising genetic, epigenetic, and stochastic components that are broadly distinguished based on characteristic timescale of change: functional genetic mutations are acquired on the order of weeks to months[8,9], transitions between epigenetic states occur on the order of hours to weeks[10], and stochastic fluctuations in protein concentrations, and other sources of intracellular noise, operate on the order of seconds to minutes[11]. Separating processes based on characteristic timescale is a common approach in physics[12] and has a long history in biology[13]. Below, we describe in detail each of these levels of heterogeneity, with precise definitions of terms that will be important for later analyses (see also Table A).

Genetic: Genomic instability is a hallmark of cancer[14,15]. For cancer cells in culture, it has been estimated that approximately five million cell divisions are necessary to acquire at least one mutation per gene[8]. With more than 20,000 genes in the genome, this amounts to one mutation every two weeks on average. These mutations can be single-nucleotide polymorphisms (SNPs), insertions/deletions (InDels), and larger structural variations. While mutations in the genome are relatively frequent, many do not have functional consequence, so-called "passenger" mutations[6]. Therefore, we make a distinction between the *genomic state* of a cell and the *genetic state*: the genomic state is the full sequence of nucleotides that make up the DNA whereas the genetic state is the subset of this sequence that contributes to a specific cellular phenotype[16,17]. In other words, *two cells may differ genomically but be genetically identical if the mutations that differentiate them occur in portions of the genome that have no functional consequence on the phenotype of interest*. As such, the timescale for generating genetic heterogeneity within a population of cancer cells can be quite slow (much slower than the acquisition of genomic heterogeneity), on the order of weeks to months[8,9]. Note that enumerating the subset of the genome that defines the genetic state can be difficult (if not impossible) in practice and depends on the phenotypic context[18]. Here, we use drug response as the phenotype of interest. Thus, the genetic state is, in principle, defined over all genes associated with drug response, which includes those involved in stress response, metabolism, cell cycle regulation, and many others.

Epigenetic: Conceptualized as a quasi-potential energy surface where local minima, or "basins of attraction," correspond to cellular phenotypes, epigenetic landscapes were first proposed by Waddington as an abstract tool for understanding phenotypic plasticity and cellular differentiation during development[19]. The genetic state of a cell sets the topography of the landscape[2,4] and intrinsic (e.g., gene expression[20]) or extrinsic[21,22] sources of noise drive transitions between phenotypes. Phenotypic state transitions (between epigenetic basins) have been observed to occur on the order of hours to weeks[23,24], meaning epigenetic diversification occurs on a much faster timescale than genetic diversification. From a molecular perspective, the epigenetic landscape is the consequence of the complex biochemical interaction networks underlying cellular function[25,26]. Complex dynamical networks can harbor multiple stable states, termed "attractors", towards which a system will tend to return (relax back) in response to small perturbations[27,28]. This property underlies epigenetic heritability: daughter cells inherit similar molecular contents to their parent; hence, they tend to remain within the region of influence of

the parental attractor. Here, we use the transcriptional state of a cell, revealed through single-cell transcriptomics, as a proxy for the epigenetic state, with the understanding that mRNA is only one lens through which to view epigenetics. Indeed, this definition of an epigenetic state, i.e., as a stable state of a complex biochemical network, differs from the traditional molecular biology definition in terms of "epigenetic marks"[29] (e.g., DNA methylation, regions of open chromatin). The two are related, however, in the sense that the same biochemical network that defines the epigenetic landscape also sets the epigenetic marks on the DNA[3] (see "*Relationship between transcriptomics and epigenetics"* below for further discussion).

<u>Stochastic</u>: Fluctuations, or noise, in intracellular species concentrations have long been recognized as a source of non-genetic heterogeneity in isogenic cell populations, first in bacteria[11,30] and then in yeast[31,32] and mammalian cells[24,33,34]. Intracellular noise can be "intrinsic", i.e., due to the probabilistic nature of biochemical interactions[35,36], or "extrinsic"[37], affecting the rates of interactions, synthesis, and degradation within a biochemical network. Intrinsic sources of noise include transcriptional bursting[38,39], translational bursting[40], and randomness in mRNA/protein degradation[41], oligomerization[42], and post-translational modification[43]. Extrinsic noise includes randomness in the distribution of molecular contents upon cell division[44,45], environmental factors such as inhomogeneities in cell culture media[46], fluctuations in temperature[47] and pH[48], and spatial variations in the microenvironment[49]. Importantly, fluctuations at the molecular level can drive probabilistic cell fate decisions[20,50–54], including division and death[55,56], at the single-cell level and phenotypic diversification at the epigenetic (population) level[34,57]. Experimentally, at the intracellular level, intrinsic and extrinsic noise are difficult to distinguish, generally requiring multiple fluorescent reporters and the ability to fine-tune the external environment[37]. Theoretically, the chemical Master Equation[58,59] (CME) is the construct upon which stochastic dynamical analyses are based. Since the CME is difficult to solve in general, a number of stochastic algorithms have been developed for simulating fluctuations at both the single cell and cell population levels[36,60] (see "*Simple growth model of stochastic birth and death"* below).

Relationship between transcriptomics and epigenetics

As mentioned above (see *"Genetic, epigenetic, and stochastic sources of tumor heterogeneity: Epigenetic"*), from a molecular standpoint, the Waddington epigenetic landscape is a consequence of the complex biochemical interaction networks underlying cellular function[25,26]. Within this framework, cell phenotypes are stable steady states, or "attractors"[27,28], of this interconnected network within cells. Each attractor is defined as a point (or cycle) within an *n*-dimensional "phase space," where each axis represents the concentration of a molecular species. The defining characteristic of an attractor is that the system will tend to return (relax back) to it in response to small perturbations (e.g., gene expression noise, uneven distribution of molecular contents upon division). It is this attractiveness that underlies epigenetic heritability: daughter cells inherit similar contents to their parent; hence, they tend to remain within the region of influence of the same attractor. Larger perturbations, however, can move a system into the region of influence of a neighboring attractor, resulting in a spontaneous phenotypic state change. This explains why epigenetic inheritance is often short lived[61].

An "epigenetic state" within this definition is thus simply the molecular state of the associated attractor (see Table A). This molecular state can be viewed through a variety of different lenses, including mass cytometry (protein level), ATAC-seq (open chromatin regions), and RNA-seq (transcriptomics). It is the latter that we have chosen in this work. However, as stated, it is important to recognize that transcriptomics serves only as one possible view, i.e., a proxy, for the epigenetic state. In molecular biology, for example, epigenetic states are usually discussed in terms of chromosomal changes[62], e.g., DNA methylation and histone modification. These are simply different views of the same epigenetic states since the biochemical network that defines the Waddington landscape also regulates the epigenetic marks on the DNA[3].

The genetic-to-epigenetic connection

In the genetic/epigenetic/stochastic (G/E/S) view of tumor heterogeneity that we utilize in this work (Figs 1 and 6 of the main text; see also *"Genetic, epigenetic, and stochastic sources of tumor heterogeneity"* above), genetics are fundamentally tied to epigenetics. To understand how, recall that an epigenetic landscape quantifies, in terms of a quasi-potential energy, the accessible molecular states that cells can occupy. The topography of the landscape depends on the molecular species present (the axes of the state-space), the biochemical processes that can occur, and the *parameters* that quantify the rates at which these processes proceed. The latter means that changes to the rate parameters can change the topography of the landscape, i.e., the depths of basins, heights of barriers, etc. As mentioned in the main text (*"Introduction"*), for processes such as protein-protein interactions, the rate parameters are directly dependent on protein structure, which is encoded in the DNA. Thus, mutations to the DNA that, e.g., alter the accessibility of a binding domain, change the values of rate parameters and, hence, the epigenetic landscape as a whole. In this way, each genetic state can be thought of as having an associated epigenetic landscape, which may have multiple basins, or cell phenotypes.

A consequence of this final point is that phenotypic differences at the transcriptomic level do not always indicate differences at the genetic level. In other words, *cells can be genetically identical yet differ transcriptomically if they occupy different basins within a common epigenetic landscape*. In theory, we should be able to distinguish between cases in which transcriptomic differences are tied to genetic variations and those in which they are not by simply comparing, across samples, parts of the genome that are relevant to the phenotype of interest (e.g., drug response). However, in practice we do not have a full accounting of all phenotypically-relevant parts of the genome, we only have the full genomic sequences, which will invariably have differences. The question is whether those differences are functionally relevant to the phenotype of interest. If not, we can consider the samples to be genetically identical, even though they are genomically distinct (see *"Genetic, epigenetic, and stochastic sources of tumor heterogeneity: Genetic"* above).

This is the rationale behind the GO term analysis we perform in this work. Four of the PC9-VU sublines (DS3, DS6, DS7, DS9) display small but measurable differences in their genomic signatures (Fig 4A–E in the main text) and appear to reside in three distinct portions of the transcriptomic space (Fig 4F). Our goal is to determine whether the small genomic differences across sublines can explain the observed transcriptomic differences. A naïve approach would be to identify genes that differentiate the sublines at the transcriptomic level and look for mutations

in those genes at the genomic level. However, it is well known that a mutation in a gene does not always alter the expression level of that gene[63]. Rather, if gene *X* encodes for a protein that acts as a transcription factor for gene *Y*, then a mutation in *X* could alter the protein structure such that it affects the expression level of *Y*. Thus, rather than making gene-by-gene comparisons of mutations and expression levels, we compare genomic variants and differentially expressed genes at the *process* level. Specifically, we compare Gene Ontology (GO) terms[64,65] for "Biological Process" (BP; 12,272 terms), "Molecular Function" (MF; 4,165 terms), and "Cellular Component" (CC; 1,740 terms) categories at the genomic and transcriptomic levels and look for correlations between them (see *"Methods"* in the main text for details). High correlation means that there is evidence that variations at the genomic level are responsible for differences at the transcriptomic level, i.e., that the subline has a distinct *genetic* state. Conversely, lack of correlation is evidence that the cells occupy a basin within a common epigenetic landscape. As a control, we perform this analysis on the cell line versions as well (VU, MGH, BR1), which we assume are genetically distinct. This provides benchmarks against which we can compare results for the sublines.

Multiple possible routes to a new genetic state in DS8

As discussed in the main text, we conclude based on our genomic and transcriptomic analyses that the DS8 subline harbors a genetic resistance mutation (different from the T790M mutation present in PC9-BR1) that emerged from the PC9-VU parental population at some point in the past. It remains an open question, however, whether this mutation occurred after or before the subline was established. If it occurred after, it could be the case that DS8 actually harbors two distinct genetic states, the original PC9-VU state and the new emergent state (S11A Fig). However, if that were the case we would expect to see some DS8 cells in the same region of transcriptomic space as PC9-VU, which we do not (Fig 4F of the main text; S6C,D Fig). One explanation could be that the emergent genetic state in DS8 outgrew the PC9-VU state, leaving behind an isogenic population (i.e., a "selective sweep"). However, there does not appear to be a discernable difference between the out-of-drug proliferation rates for DS8 and the other sublines (Fig 2C of the main text), which would seem to discount this possibility. We cannot entirely preclude this prospect, however, as it is possible that the difference in proliferation rates is simply too slight to detect in our current data.

Conversely, if the mutation in DS8 arose prior to the subline being established, we would expect some mutant cells to remain within the PC9-VU parental population. We do, in fact, see a small number of PC9-VU cells in the region of transcriptomic space where DS8 cells reside (compare Fig 3F and 4F of the main text). Interestingly, we also see some DS9 cells in this region (but not DS7, despite the significant overlap between the two populations; Fig 4F of the main text and S6C,D Fig). This could indicate that DS9 actually comprises two epigenetic basins, a deep one where most of the cells reside and a shallow one in the region of transcriptomic space where DS8 cells reside. It is possible that PC9-VU cells transition frequently between these two basins and that at one point in the past one cell in the shallow basin randomly acquired a genetic mutation that caused it to get "locked into" that basin. A progeny of that cell could have been isolated to start the DS8 subline (S11B Fig). This scenario would be consistent with the findings of Shaffer et al.[66], although without the selective pressure presumed to drive the mutation in

their case. However, if this is the case, given that DS8 proliferates at essentially the same rate as the other sublines in the absence of drug, why are those cells so rare (~2%) within the PC9-VU parental population? One would expect that population to grow out in the same way that DS8 did. One explanation might be that cell-cell interactions[67] destabilize the mutant cells when in culture together with PC9-VU parental cells. This is an intriguing idea that would require additional experimentation to verify, perhaps using DNA barcoding to track lineages within co-cultured populations.

Finally, there is also the added complication that DS8 exhibits a bimodal DIP rate distribution under EGFRi, which we conclude from our stochastic simulation analysis (Fig 5E–H of the main text) indicates that DS8 harbors (at least) two distinct cell states. As discussed in the main text, the model is agnostic as to whether these two states are genetically identical or not, it merely requires they have distinct DIP rates. Thus, it does not preclude the possibility that DS8 is isogenic, as would be the case in two of the scenarios discussed above (*i.* mutation after subline establishment, followed by selective sweep; *ii.* mutation prior to subline establishment). However, the region of transcriptomic space in which DS8 cells reside is small compared to the region occupied by PC9-VU and there are no obvious subpopulations present (Fig 4F of the main text). It is also difficult to reconcile why the left mode of the DS8 DIP rate distribution overlaps so significantly with the PC9-VU parental and subline distributions (Fig 2D of the main text) despite the lack of overlap in transcriptomic space. These remain open questions that we hope to address in future investigations. We emphasize, however, that none of the issues raised here changes our main conclusion presented in the main text that DS8 harbors a novel genetic resistance mutation that emerged from PC9-VU at some point in the past in the absence of selective pressures (Fig 6 of the main text).

Table A. Glossary of relevant terms.

Phenotype	An observable property of a cell population, either in the absence or in response to a perturbation. In this work, our phenotype of interest is response to EGFR inhibition, which we quantify in terms of single-cell transcriptomic signatures (no drug) and drug-induced proliferation (DIP) rates. Since population-level quantities like DIP rate are driven by intracellular regulatory networks, we consider transcriptomic signatures and DIP rates to be two different presentations of the same phenotype.
Genomic state	The full sequence of nucleotides that comprise a genome. With four types of nucleotides (A, C, T, G) and ~3.2 billion nucleotides per genome, the number of possible nucleotide configurations is massive (effectively infinite, even when excluding non-viable configurations). It is therefore exceedingly rare for two cells to have the same genomic state. In this work, we quantify genomic states using whole-exome sequencing.
Functional genomic regions	Locations in the DNA sequence that encode for genes that impact the phenotype of interest in some quantifiable way.
Genetic state	The sequence of nucleotides comprising only functional genomic regions of the DNA. Because the scope is much more limited, the number of possible genetic states available to a cell is significantly smaller than the number of genomic states, i.e., many cells, up to and including an entire cell population, may share the same genetic state.
Molecular state	The concentrations of all biochemical species (mRNAs, proteins, small molecules, etc.) associated with a phenotype of interest. Because copy numbers can be large and fluctuate due to intrinsic and extrinsic sources of noise, it is exceedingly rare for two cells to have the same molecular state. In this work, we quantify molecular states using single-cell transcriptomics.
Epigenetic landscape	A conceptual and mathematical construct that assigns a "quasi-potential energy" to all possible molecular states of a cell. Fundamentally, the epigenetic landscape is the consequence of the complex biochemical interaction networks that underlie the phenotype of interest. Importantly, each genetic state has an associated epigenetic landscape. Mutations in the DNA can thus change the topography of the landscape.

Epigenetic state	Often used interchangeably with “phenotypic state,” any heritable state of a cell that is not encoded in the DNA. Within the framework of the epigenetic landscape, an epigenetic state is the collection of states associated with each local minima, or “basin of attraction.” Cells within basis of attraction will tend to stay within the vicinity of the basin in response to small perturbations. This is the basis for epigenetic heritability since daughter cells tend to have similar molecular contents as their parent, i.e., they inherit the same basin of attraction. Conceptually, an epigenetic landscape can be seen as a collection of epigenetic states separated by barriers. Traversals of these barriers correspond to phenotypic state transitions. Importantly, cells can differ in their molecular states (e.g., due to fluctuations in gene expression) but still occupy the same epigenetic state.

Table B. List of genes associated with mutation heatmaps in Figs 3D and 4D.

Index	Gene	Index	Gene	Index	Gene
1	BHLHA15	35	IL32	69	AC092647.5
2	NCR2	36	SOX21	70	ARID1B
3	TMEM158	37	GDF11	71	CNPY3
4	JAGN1	38	MECP2	72	FOXD1
5	DLX4	39	VKORC1L1	73	TPPP
6	RAD51B	40	SMAP1	74	PRDM8
7	FAU	41	XBP1	75	OLIG1
8	AGAP3	42	RAPH1	76	DBNDD2
9	KRTAP10-6	43	MMP17	77	MYL1
10	CGREF1	44	TERF1	78	LRP1B
11	RPS15	45	POU3F2	79	CCDC74A
12	TUBB8P12	46	BCHE	80	ZNF257
13	HCRT	47	POU3F3	81	YY1
14	SKA3	48	ZFP36L2	82	FOXN3
15	OR8G1	49	ZNF787	83	PCDH9
16	GPR37L1	50	BCL6B	84	MMP1
17	BHLHE22	51	TDG	85	RRP8
18	RRAGD	52	IGSF21	86	TCHH
19	VEGFC	53	VCX3B	87	PRAMEF6
20	NAP1L5	54	OR2A1		
21	SOX10	55	SLC35F1		
22	IGFBP2	56	CADM4		
23	ADRA2B	57	NPIPB3		
24	SOX11	58	FAM155A		
25	FAM98C	59	PHLDA1		
26	CEBPA	60	RAB21		
27	PHOSPHO1	61	OR2T35		
28	PHGR1	62	FOXO6		
29	BRI3BP	63	POU3F1		
30	CARNMT1	64	FMR1NB		
31	TAF11L2	65	ASTN2		
32	EPB41L1	66	SNX31		
33	CAPNS1	67	MNX1		
34	ACSM2A	68	ZNF713		

Table C. Rate parameters used for monoclonal and polyclonal growth models.
Ranges are shown in square brackets, units are in h^{-1}, $k_{DIP} = k_{div}-k_{dth}$. Note that DIP=$k_{DIP}$/ln(2).

Subline / Parameter	DS1	DS3	DS4	DS6	DS7	DS8	DS9
$k_{div,pre-drug}$ / ln(2)	0.04	0.04)	0.04	0.04	0.04	0.04	0.04
$k_{DIP,pre-drug}$ / ln(2)	0.035	0.035	0.035	0.035	0.035	0.035	0.035
$k_{div1,post-drug}$ / ln(2)	[0.0144, 0.115]	[0.0144, 0.115]	[0.0144, 0.115]	[0.0144, 0.115]	[0.0144, 0.115]	[0.0144, 0.115]	[0.0144, 0.115]
$k_{DIP1,post-drug}$ / ln(2)	[0.000721, 0.00361]	[-0.00216, 0.000721]	[0.00361, 0.00649]	[-0.000721, 0.00216]	[0.00144, 0.00433]	[0.000721, 0.00216]	[0, 0.00289]
$k_{div2,post-drug}$ / ln(2)	N/A	N/A	N/A	N/A	N/A	[0.0144, 0.115]	N/A
$k_{DIP2,post-drug}$ / ln(2)	N/A	N/A	N/A	N/A	N/A	[0.00793, 0.0108]	N/A

Supplementary References

1. Jia D, Jolly MK, Kulkarni P, Levine H. Phenotypic plasticity and cell fate decisions in cancer: Insights from dynamical systems theory. *Cancers (Basel)*. 2017;9(7):1-19. doi:10.3390/cancers9070070
2. Huang S. Genetic and non-genetic instability in tumor progression: link between the fitness landscape and the epigenetic landscape of cancer cells. *Cancer Metastasis Rev*. 2013;32(3-4):423-448. doi:10.1007/s10555-013-9435-7
3. Huang S. Non-genetic heterogeneity of cells in development: more than just noise. *Development*. 2009;136(23):3853-3862. doi:10.1242/dev.035139
4. Marusyk A, Polyak K. Tumor heterogeneity: Causes and consequences. *Biochim Biophys Acta - Rev Cancer*. 2010;1805(1):105-117. doi:10.1016/J.BBCAN.2009.11.002
5. Marusyk A, Almendro V, Polyak K. Intra-tumour heterogeneity: a looking glass for cancer? *Nat Rev Cancer*. 2012;12(5):323-334. doi:10.1038/nrc3261
6. Hinohara K, Polyak K. Intratumoral heterogeneity: more than just mutations. *Trends Cell Biol*. April 2019. doi:10.1016/j.tcb.2019.03.003
7. Wang J, Zhang K, Xu L, Wang E. Quantifying the Waddington landscape and biological paths for development and differentiation. *Proc Natl Acad Sci U S A*. 2011;108(20):8257-8262. doi:10.1073/pnas.1017017108
8. Jackson AL, Loeb LA. The mutation rate and cancer. *Genetics*. 1998;148(4):1483-1490. doi:10.1073/pnas.93.25.14800
9. Lawrence MS, Stojanov P, Polak P, et al. Mutational heterogeneity in cancer and the search for new cancer-associated genes. *Nature*. 2013;499(7457):214-218. doi:10.1038/nature12213
10. Sasai M, Kawabata Y, Makishi K, Itoh K, Terada TP. Time scales in epigenetic dynamics and phenotypic heterogeneity of embryonic stem cells. *PLoS Comput Biol*. 2013;9(12). doi:10.1371/journal.pcbi.1003380
11. Elowitz MB, Levine AJ, Siggia ED, Swain PS. Stochastic gene expression in a single cell. *Science (80-)*. 2002;297(5584):1183-1186. doi:10.1126/science.1070919
12. ’t Hooft G, Vandoren S, ’t Hooft S. *Time in Powers of Ten : Natural Phenomena and Their Timescales.* World Scientific Publishing Company; 2014.
13. Gunawardena J. Time-scale separation – Michaelis and Menten’s old idea, still bearing fruit. *FEBS J*. 2014;281(2):473-488. doi:10.1111/febs.12532
14. Hanahan D, Weinberg RA. The hallmarks of cancer. *Cell*. 2000;100(1):57-70. doi:10.1016/s0092-8674(00)81683-9
15. Hanahan D, Weinberg RAA. Hallmarks of cancer: the next generation. *Cell*. 2011;144(5):646-674. doi:10.1016/j.cell.2011.02.013
16. Weitzel JN, Blazer KR, MacDonald DJ, Culver JO, Offit K. Genetics, genomics, and cancer risk assessment: State of the art and future directions in the era of personalized medicine. *CA Cancer J Clin*. 2011;61(5):327-359. doi:10.3322/caac.20128
17. Balmain A, Gray J, Ponder B. The genetics and genomics of cancer. *Nat Genet*. 2003;33(3S):238-244. doi:10.1038/ng1107
18. McClellan J, King MC. Genetic heterogeneity in human disease. *Cell*. 2010;141(2):210-217. doi:10.1016/j.cell.2010.03.032

19. Waddington CH. Canalization of development and the inheritance of acquired characters. *Nature*. 1942;150(3811):563-565. doi:10.1038/150563a0
20. Raj A, van Oudenaarden A. Nature, nurture, or chance: stochastic gene expression and its consequences. *Cell*. 2008;135(2):216-226. doi:10.1016/j.cell.2008.09.050
21. Kar S, Baumann WT, Paul MR, Tyson JJ. Exploring the roles of noise in the eukaryotic cell cycle. *Proc Natl Acad Sci U S A*. 2009;106(16):6471-6476. doi:10.1073/pnas.0810034106
22. Niepel M, Spencer SL, Sorger PK. Non-genetic cell-to-cell variability and the consequences for pharmacology. *Curr Opin Chem Biol*. 2009;13(5-6):556-561. doi:10.1016/J.CBPA.2009.09.015
23. Paudel BB, Harris LA, Hardeman KN, et al. A nonquiescent “idling” population state in drug-treated, BRAF-mutated melanoma. *Biophys J*. 2018;114(6):1499-1511. doi:10.1016/j.bpj.2018.01.016
24. Sharma S V, Lee DY, Li B, et al. A chromatin-mediated reversible drug-tolerant state in cancer cell subpopulations. *Cell*. 2010;141(1):69-80. doi:10.1016/j.cell.2010.02.027
25. Moris N, Pina C, Arias AM. Transition states and cell fate decisions in epigenetic landscapes. *Nat Rev Genet*. 2016;17(11):693-703. doi:10.1038/nrg.2016.98
26. Huang S. The molecular and mathematical basis of Waddington’s epigenetic landscape: A framework for post-Darwinian biology? *BioEssays*. 2012;34(2):149-157. doi:10.1002/bies.201100031
27. Zhou JX, Aliyu MDS, Aurell E, Huang S. Quasi-potential landscape in complex multi-stable systems. *J R Soc Interface*. 2012;9(77):3539-3553. doi:10.1098/rsif.2012.0434
28. Britton N. *Essential Mathematical Biology*. London: Springer-Verlag; 2005. doi:10.1007/978-1-4471-0049-2
29. Ptashne M. On the use of the word “epigenetic.” *Curr Biol*. 2007. doi:10.1016/j.cub.2007.02.030
30. Thattai M, Van Oudenaarden A. Stochastic gene expression in fluctuating environments. *Genetics*. 2004;167(1):523-530. doi:10.1534/genetics.167.1.523
31. Muzzey D, Gomez-Uribe CA, Mettetal JT, van Oudenaarden A. A systems-level analysis of perfect adaptation in yeast osmoregulation. *Cell*. 2009;138(1):160-171. doi:10.1016/j.cell.2009.04.047
32. Neuert G, Munsky B, Tan RZ, Teytelman L, Khammash M, Oudenaarden A Van. Systematic identification of signal-activated stochastic gene regulation. *Science (80-)*. 2013;339(6119):584-587. doi:10.1126/science.1231456
33. Magklara A, Lomvardas S. Stochastic gene expression in mammals: lessons from olfaction. *Trends Cell Biol*. 2013;23(9):449-456. doi:10.1016/j.tcb.2013.04.005
34. Gupta PB, Fillmore CM, Jiang G, et al. Stochastic state transitions give rise to phenotypic equilibrium in populations of cancer cells. *Cell*. 2011;146(4):633-644. doi:10.1016/j.cell.2011.07.026
35. Gillespie DT. The chemical Langevin equation. *J Chem Phys*. 2000;113(1):297. doi:10.1063/1.481811
36. Gillespie DT. Stochastic simulation of chemical kinetics. *Annu Rev Phys Chem*. 2007;58(1):35-55. doi:10.1146/annurev.physchem.58.032806.104637
37. Hilfinger A, Paulsson J. Separating intrinsic from extrinsic fluctuations in dynamic biological systems. *Proc Natl Acad Sci U S A*. 2011;108(29):12167-12172.

doi:10.1073/pnas.1018832108
38. Friedman N, Cai L, Xie XS. Linking stochastic dynamics to population distribution: An analytical framework of gene expression. *Phys Rev Lett*. 2006;97(16):1-4. doi:10.1103/PhysRevLett.97.168302
39. Mcadams HH, Arkin A. Stochastic mechanisms in gene expression. *Proc Natl Acad Sci U S A*. 1997;94(3):814-819. doi:10.1073/pnas.94.3.814
40. Sigal A, Milo R, Cohen A, et al. Variability and memory of protein levels in human cells. *Nature*. 2006;444(7119):643-646. doi:10.1038/nature05316
41. El-Samad H, Khammash M. Regulated degradation is a mechanism for suppressing stochastic fluctuations in gene regulatory networks. *Biophys J*. 2006;90(10):3749-3761. doi:10.1529/biophysj.105.060491
42. Alarcón T, Page KM. Stochastic models of receptor oligomerization by bivalent ligand. *J R Soc Interface*. 2006;3(9):545-559. doi:10.1098/rsif.2006.0116
43. Ladbury JE, Arold ST. Noise in cellular signaling pathways: causes and effects. *Trends Biochem Sci*. 2012;37(5):173-178. doi:10.1016/j.tibs.2012.01.001
44. Huh D, Paulsson J. Random partitioning of molecules at cell division. *Proc Natl Acad Sci*. 2011;108(36):15004-15009. doi:10.1073/pnas.1013171108
45. Huh D, Paulsson J. Non-genetic heterogeneity from stochastic partitioning at cell division. *Nat Genet*. 2011;43(2):95-100. doi:10.1038/ng.729
46. Hardeman KN, Peng C, Paudel BB, et al. Dependence on glycolysis sensitizes BRAF-mutated melanomas for increased response to targeted BRAF inhibition. *Sci Rep*. 2017;7:42604. doi:10.1038/srep42604
47. Charlebois DA, Hauser K, Marshall S, Balázsi G. Multiscale effects of heating and cooling on genes and gene networks. *Proc Natl Acad Sci U S A*. 2018;115(45):E10797-E10806. doi:10.1073/pnas.1810858115
48. Webb BA, Chimenti M, Jacobson MP, Barber DL. Dysregulated pH: A perfect storm for cancer progression. *Nat Rev Cancer*. 2011;11(9):671-677. doi:10.1038/nrc3110
49. Lee CF, Brangwynne CP, Gharakhani J, Hyman AA, Jülicher F. Spatial organization of the cell cytoplasm by position-dependent phase separation. *Phys Rev Lett*. 2013;111(8):1-5. doi:10.1103/PhysRevLett.111.088101
50. Arkin A, Ross J, McAdams HH. Stochastic kinetic analysis of developmental pathway bifurcation in phage lambda-infected Escherichia coli cells. *Genetics*. 1998;149(4):1633-1648. https://www.genetics.org/content/149/4/1633.full.
51. McAdams HH, Arkin A. It's a noisy business! Genetic regulation at the nanomolar scale. *Trends Genet*. 1999;15(2):65-69. doi:10.1016/s0168-9525(98)01659-x
52. Losick R, Desplan C. Stochasticity and cell fate. *Science*. 2008;320(5872):65-68. doi:10.1126/science.1147888
53. Samoilov MS, Price G, Arkin AP. From fluctuations to phenotypes: the physiology of noise. *Sci STKE*. 2006;2006(366):re17. doi:10.1126/stke.3662006re17
54. Rao C V., Wolf DM, Arkin AP. Control, exploitation and tolerance of intracellular noise. *Nature*. 2002;420(6912):231-237. doi:10.1038/nature01258
55. Tyson DR, Garbett SP, Frick PL, Quaranta V. Fractional proliferation: a method to deconvolve cell population dynamics from single-cell data. *Nat Methods*. 2012;9(9):923-928. doi:10.1038/nmeth.2138

56. Harris LA, Frick PL, Garbett SP, et al. An unbiased metric of antiproliferative drug effect in vitro. *Nat Methods*. 2016;13(6):497-500. doi:10.1038/nmeth.3852
57. Gupta PB, Pastushenko I, Skibinski A, Blanpain C, Kuperwasser C. Phenotypic plasticity: driver of cancer initiation, progression, and therapy resistance. *Cell Stem Cell*. 2019;24(1):65-78. doi:10.1016/j.stem.2018.11.011
58. McQuarrie DA. Stochastic approach to chemical kinetics. *J Appl Probab*. 1967;4(3):413-478. doi:10.2307/3212214
59. Gillespie DT. A rigorous derivation of the chemical master equation. *Phys A Stat Mech its Appl*. 1992;188(1-3):404-425. doi:10.1016/0378-4371(92)90283-V
60. Li H, Cao Y, Petzold LR, Gillespie DT. Algorithms and software for stochastic simulation of biochemical reacting systems. *Biotechnol Prog*. 2008;24(1):56-61. doi:10.1021/bp070255h
61. D'Urso A, Brickner JH. Mechanisms of epigenetic memory. *Trends Genet*. 2014;30(6):230-236. doi:10.1016/j.tig.2014.04.004
62. Berger SL, Kouzarides T, Shiekhattar R, Shilatifard A. An operational definition of epigenetics. *Genes Dev*. 2009;23(7):781-783. doi:10.1101/gad.1787609
63. Gerstung M, Pellagatti A, Malcovati L, et al. Combining gene mutation with gene expression data improves outcome prediction in myelodysplastic syndromes. *Nat Commun*. 2015;6(1):5901. doi:10.1038/ncomms6901
64. Carbon S, Douglass E, Dunn N, et al. The Gene Ontology Resource: 20 years and still GOing strong. *Nucleic Acids Res*. 2019;47(D1):D330-D338. doi:10.1093/nar/gky1055
65. Ashburner M, Ball CA, Blake JA, et al. Gene Ontology: tool for the unification of biology. *Nat Genet*. 2000;25(1):25-29. doi:10.1038/75556
66. Shaffer SM, Dunagin MC, Torborg SR, et al. Rare cell variability and drug-induced reprogramming as a mode of cancer drug resistance. *Nature*. 2017;546(7658):431-435. doi:10.1038/nature22794
67. Harris LA, Beik S, Ozawa PMM, Jimenez L, Weaver AM. Modeling heterogeneous tumor growth dynamics and cell–cell interactions at single-cell and cell-population resolution. *Curr Opin Syst Biol*. 2019;17:24-34. doi:10.1016/J.COISB.2019.09.005
