# Supplementary figures and images for: An in vitro model of tumor heterogeneity resolves genetic, epigenetic, and stochastic sources of cell state variability
Source: PLoS Biol. 2021 Jun 1;19(6):e3000797. doi: 10.1371/journal.pbio.3000797 (PMC8195356; doi:10.1371/journal.pbio.3000797)

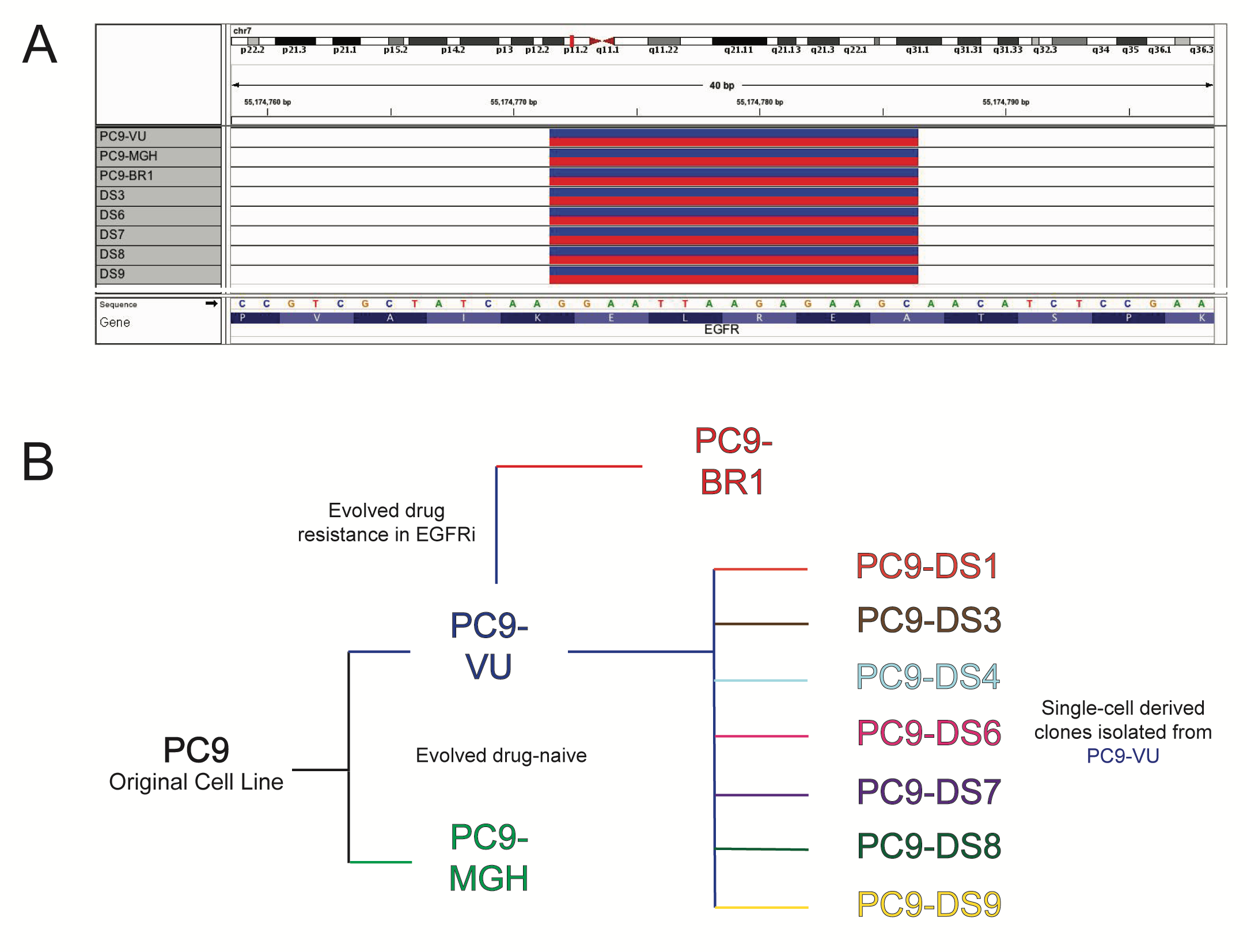

Supplement: S1 Fig — (A) Identification of canonical EGFR-ex19del in PC9 cell line family members. A screenshot from the IGV is shown. Red corresponds to potential deletions and blue to potential insertions. The data underlying this image can be found in the Sequence Read Archive (ncbi.nlm.nih.gov/sra) at accession #PRJNA632351. (B) PC9 cell line family tree. Two versions the PC9 cell line were maintained separately in culture at 2 different institutions (VU and MGH). A resistant cell line (PC9-BR1) was derived from PC9-VU by dose escalation in the EGFRi afatinib. Several DS were also single-cell isolated from PC9-VU. Colors are consistent with data visualizations in main and supplementary figures. DS, discrete subline; EGFR, epidermal growth factor receptor; EGFRi, EGFR inhibitor; IGV, Integrative Genomics Viewer; MGH, Massachusetts General Hospital; VU, Vanderbilt University. (TIF) [file pbio.3000797.s002.tif]

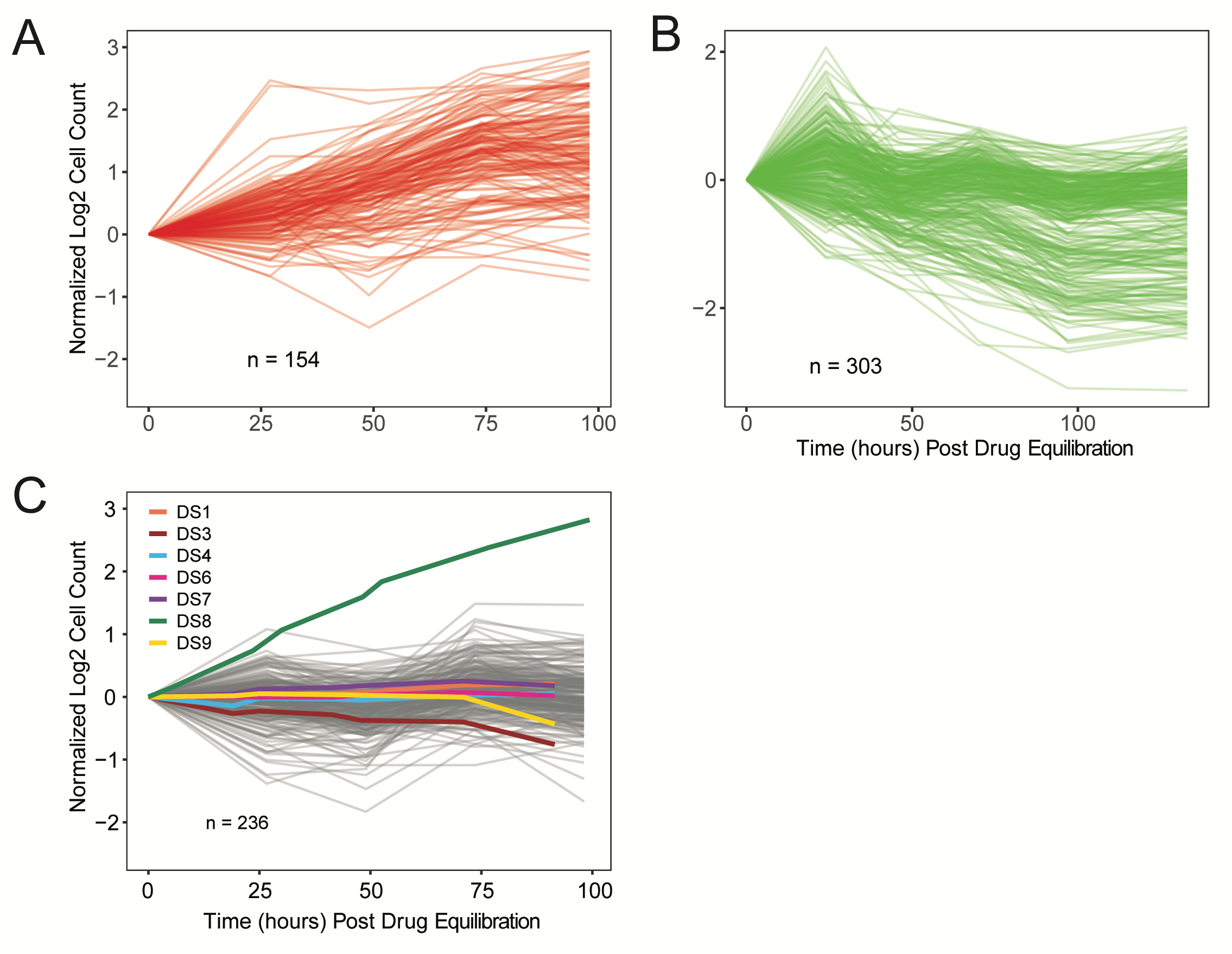

Supplement: S2 Fig — (A) PC9-MGH treated with erlotinib. (B) PC9-BR1 treated with erlotinib. All trajectories in A and B are normalized to approximately 72 h postdrug treatment. (C) PC9-VU treated with erlotinib. Trajectories for the parental line (gray) and the discrete sublines (colors) are plotted together for comparison. All subline trajectories, except DS8, are normalized to approximately 125 h post-erlotinib treatment; DS8 was normalized to the time of treatment because it was resistant and reached confluency during the course of the experiment. For the sublines, means of time point replicates are plotted. In all cases, number of colonies (n) are noted within the plots. The data underlying this figure can be found in github.com/QuLab-VU/GES_2021. cFP, clonal fractional proliferation. (TIF) [file pbio.3000797.s003.tif]

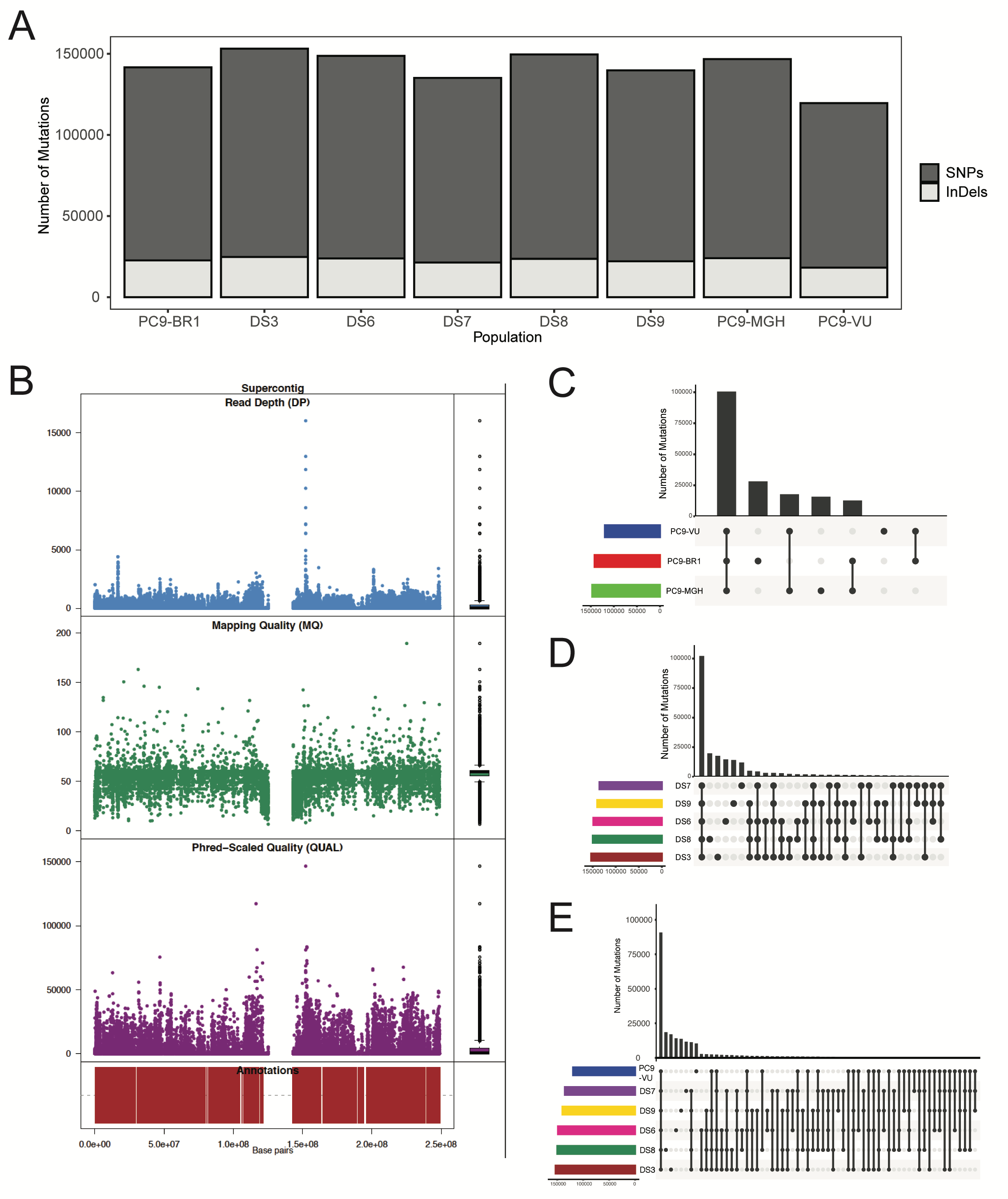

Supplement: S3 Fig — (A) Total number of mutations identified through variant calling compared to hg38 reference genome. Mutations are separated into substitutions, specifically SNPs and InDels. (B) Sequencing quality metrics for the PC9 cell line family (considered together as one group). DP is a measure of sequence coverage; MQ details how well the sequencing reads are mapped to the reference genome; QUAL is a score developed for Phred base calling that measures the confidence in called variants based on sequencing error probabilities; variant count is a reflection of the variants per site identified over small sections (windows) of the reference genome. (C–E) Quantified Venn diagram (i.e., UpSet plot) of unique, and intersections of, mutations in (C) cell line versions, (D) PC9-VU sublines, and (E) PC9-VU sublines and parental. The data underlying this figure can be found in github.com/QuLab-VU/GES_2021. InDel, insertion/deletion; SNP, single nucleotide polymorphism; WES, whole exome sequencing. (TIF) [file pbio.3000797.s004.tif]

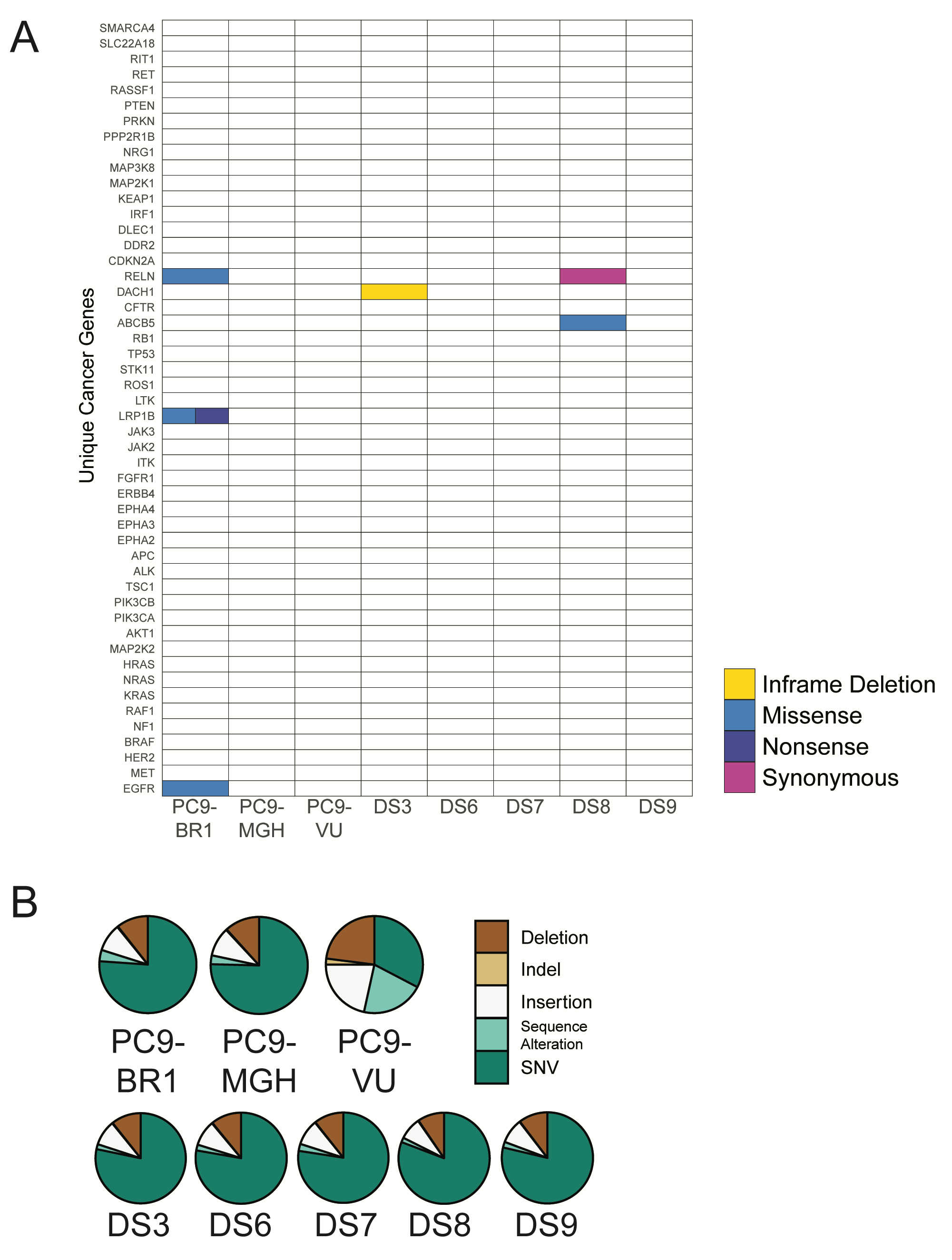

Supplement: S4 Fig — (A) Mutational differences between PC9 cell line family members for a literature-curated set of cancer-associated genes implicated in lung cancer (see Materials and methods). Heatmap elements are colored based on type of mutation. (B) Mutation class pie charts. The data underlying this figure can be found in github.com/QuLab-VU/GES_2021. Indel, insertion/deletion; SNV, single nucleotide variant. (TIF) [file pbio.3000797.s005.tif]

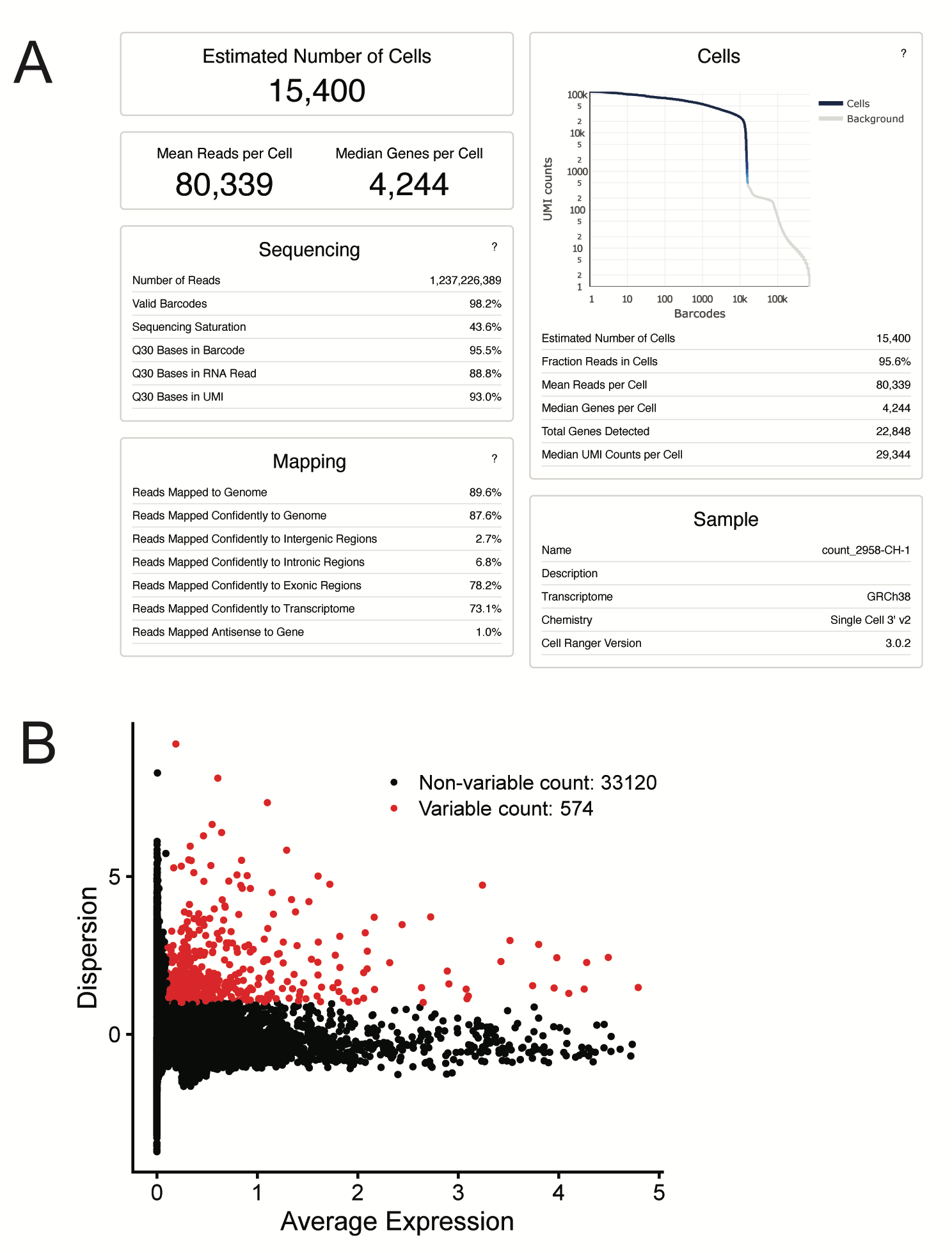

Supplement: S5 Fig — (A) Cell Ranger (support.10xgenomics.com/single-cell-gene-expression/software) output file detailing metrics of sequencing run (quality, mapping, barcode identification, etc.). The data underlying this image can be found in the Gene Expression Omnibus (ncbi.nlm.nih.gov/geo) data repository at accession #GSE150084. (B) Feature identification for genes that transcriptomically differentiate PC9 cell line family members. Variable genes are projected on a plot of dispersion vs. average gene expression and genes that pass a feature selection threshold are shown in red (0.1<average gene expression<8, log variance-to-mean ratio>1; 574 genes). The data underlying this plot can be found in github.com/QuLab-VU/GES_2021. scRNA-seq, single-cell RNA sequencing; UMI, unique molecular identifier. (TIF) [file pbio.3000797.s006.tif]

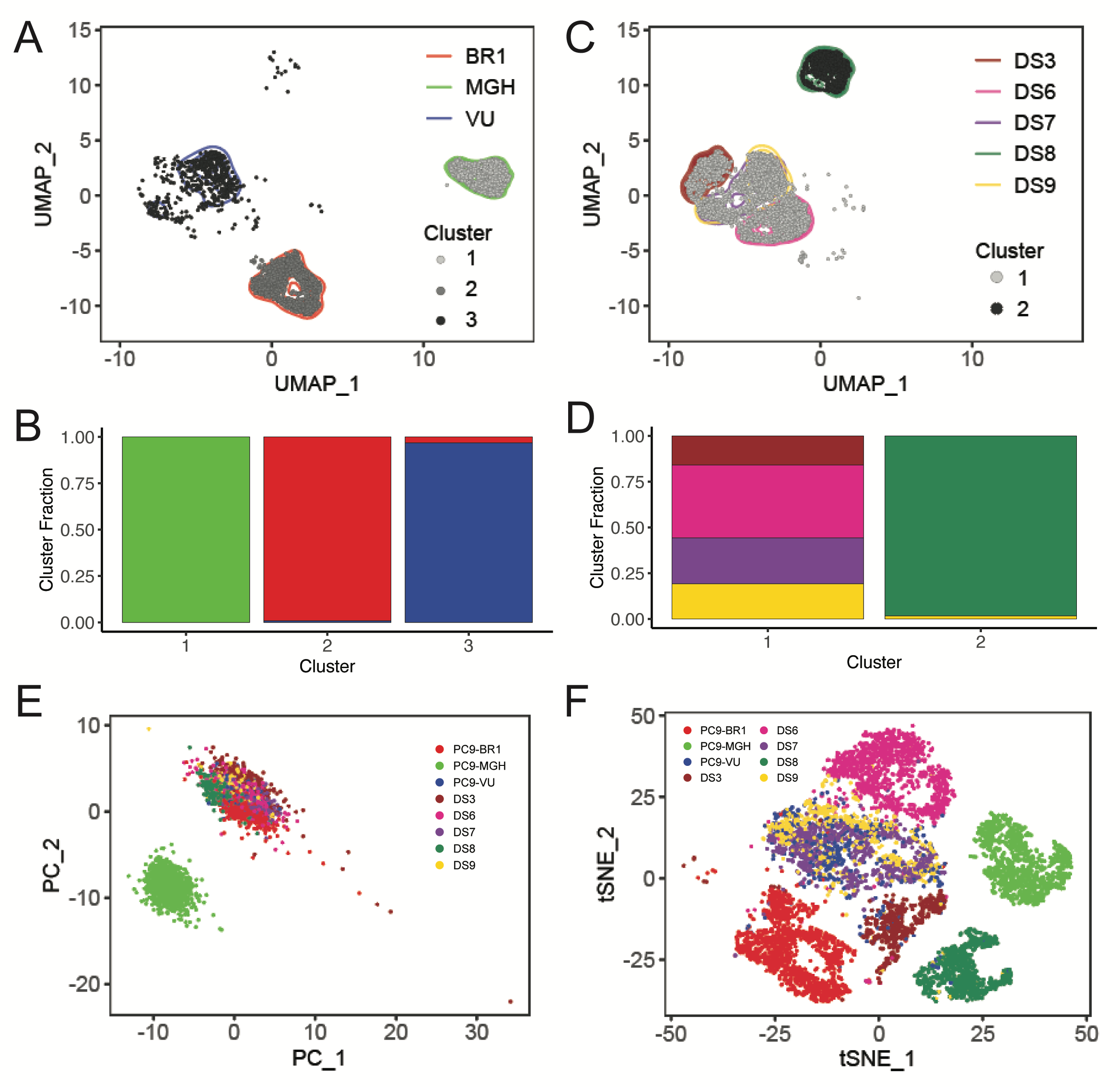

Supplement: S6 Fig — (A) Clustering of cell line versions. Number of clusters (3) was defined based on majority rule from a consensus of 30 indices. Ward’s minimum variance method was used. (B) Quantification of cluster fraction by cell line version. (C) Same as A but for sublines. Two clusters were found to be the consensus. (D) Same as B but for sublines. (E) PCA visualization of single-cell transcriptomes. (F) t-SNE visualization of single-cell transcriptomes. The data underlying this figure can be found in github.com/QuLab-VU/GES_2021. PCA, principal component analysis; scRNA-seq, single-cell RNA sequencing; t-SNE, t-distributed Stochastic Neighbor Embedding. (TIF) [file pbio.3000797.s007.tif]

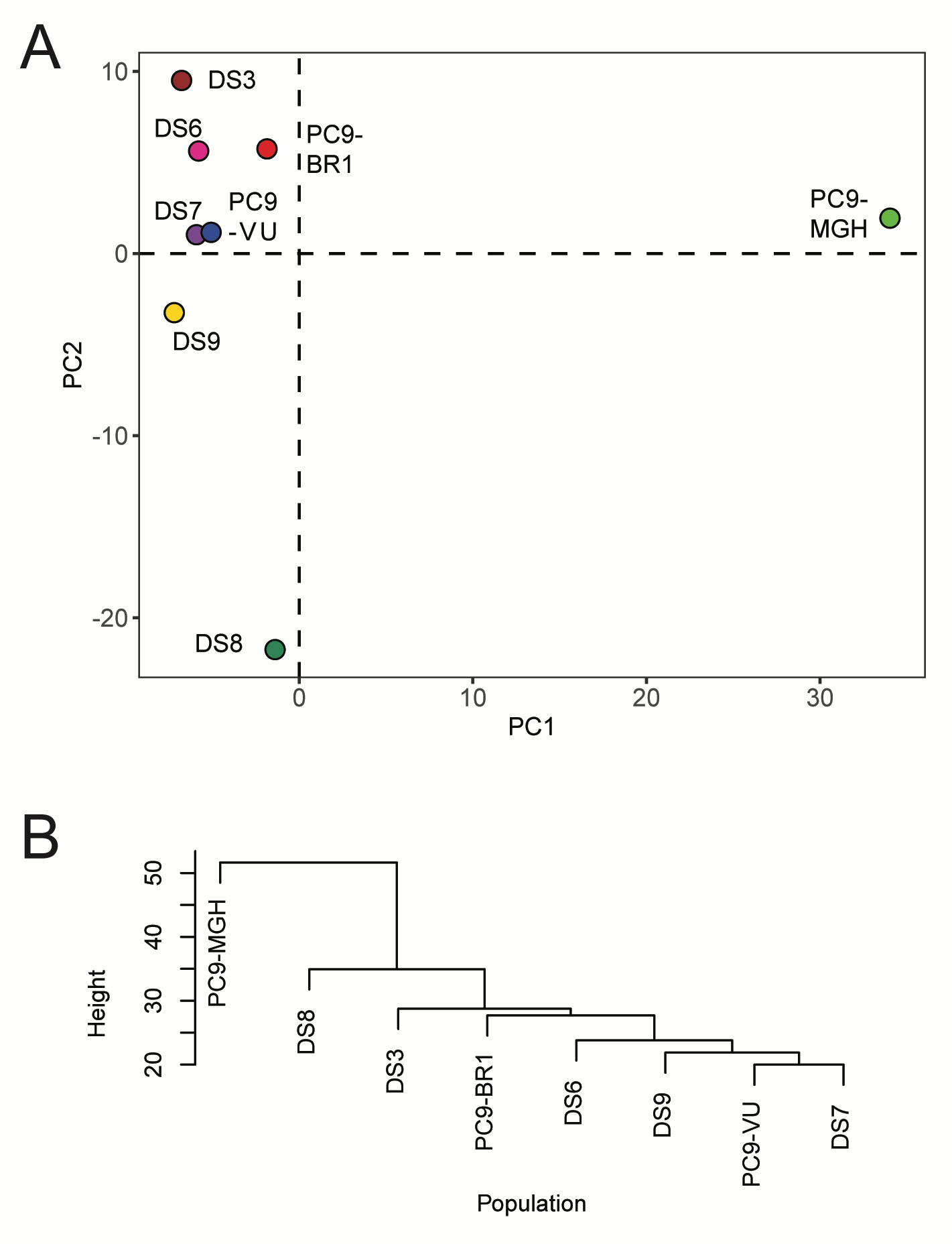

Supplement: S7 Fig — (A) PCA of single-replicate normalized RNA-seq count data. (B) Hierarchical clustering of RNA-seq normalized count data. Clustering was performed on the pairwise Euclidian distance matrix created from the relative log transformed gene counts using the Ward’s minimum variance method. The data underlying this figure can be found in github.com/QuLab-VU/GES_2021. PCA, principal component analysis; RNA-seq, RNA sequencing. (TIF) [file pbio.3000797.s008.tif]

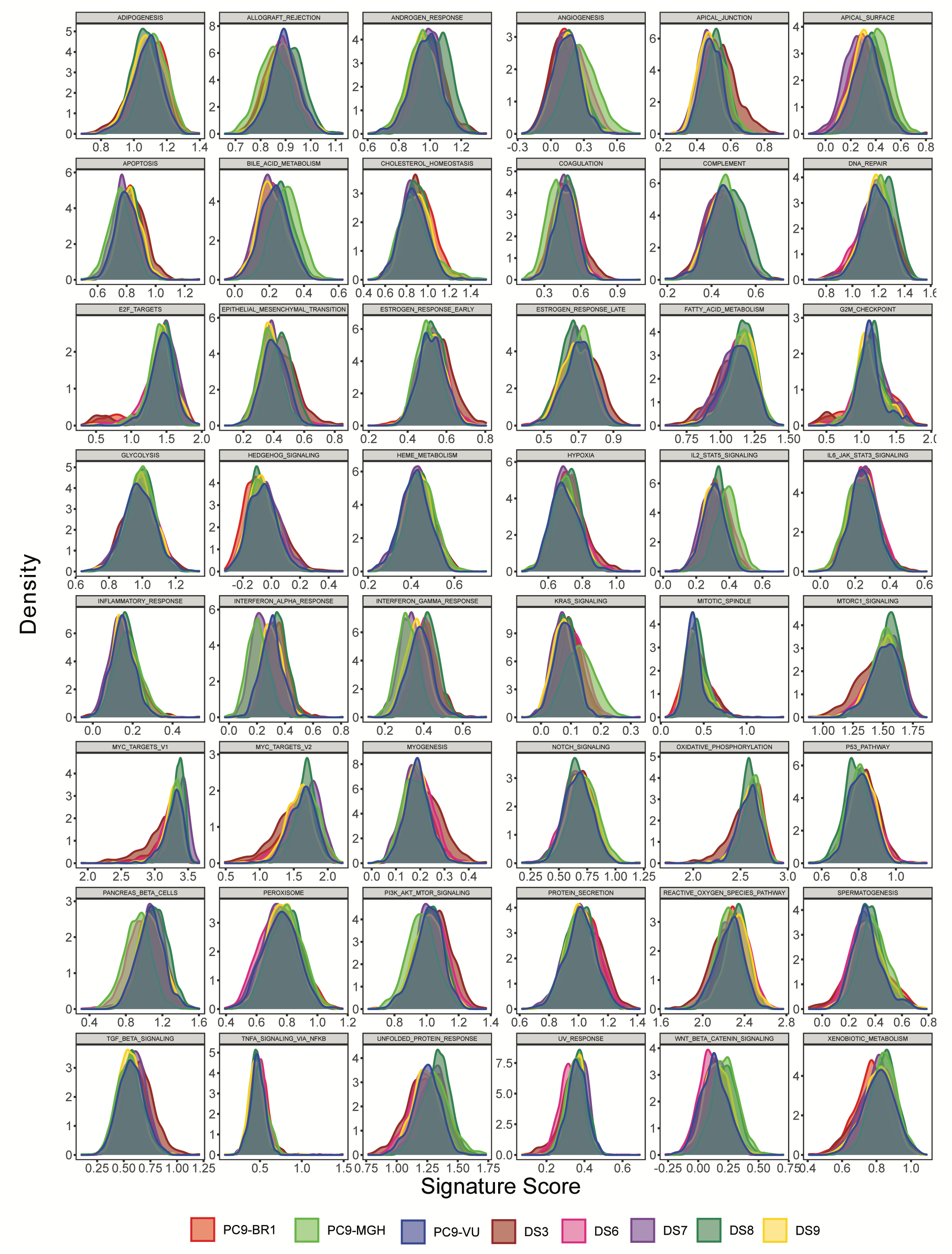

Supplement: S8 Fig — Single-cell gene expression matrix and MSigDB hallmark gene signatures were input to create a signature score for each cell. Scores were totaled for each population across each hallmark and plotted as a density distribution. All 50 hallmark signatures were sampled. Note that “KRAS signaling” and “UV response” had hallmark signatures for both up- and down-regulated. We condensed these 4 signatures into 2, leaving 48 hallmark signatures total. The data underlying this figure can be found in github.com/QuLab-VU/GES_2021. MSigDB, molecular signatures database. (TIF) [file pbio.3000797.s009.tif]

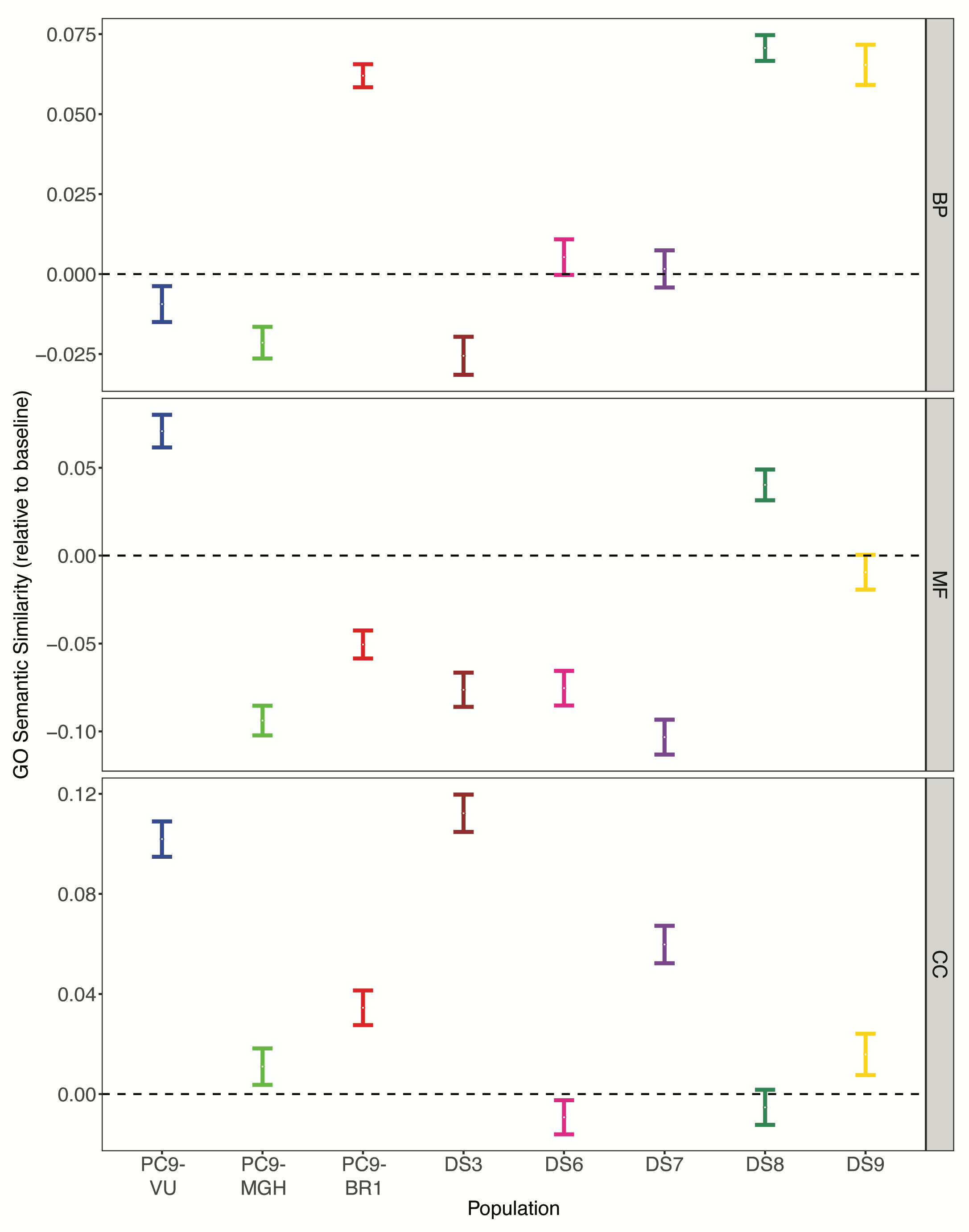

Supplement: S9 Fig — Significantly enriched GO terms for each data modality pair (IMPACT mutations and DEGs) were compared for each cell line family member for each GO type (BP, MF, and CC). The top 1,000 similarity scores within each pair were compiled into a distribution to calculate a median (white circle) and 95% confidence interval (error bars). Scores are plotted relative to a baseline, defined as the median + one standard deviation of simulated distributions (dashed lines). Simulated score distributions were calculated based on random gene lists of identical lengths to the experimental gene lists (see Materials and methods). The data underlying this figure can be found in github.com/QuLab-VU/GES_2021. BP, Biological Process; CC, Cellular Component; DEG, differentially expressed gene; GO, Gene Ontology; MF, Molecular Function. (TIF) [file pbio.3000797.s010.tif]

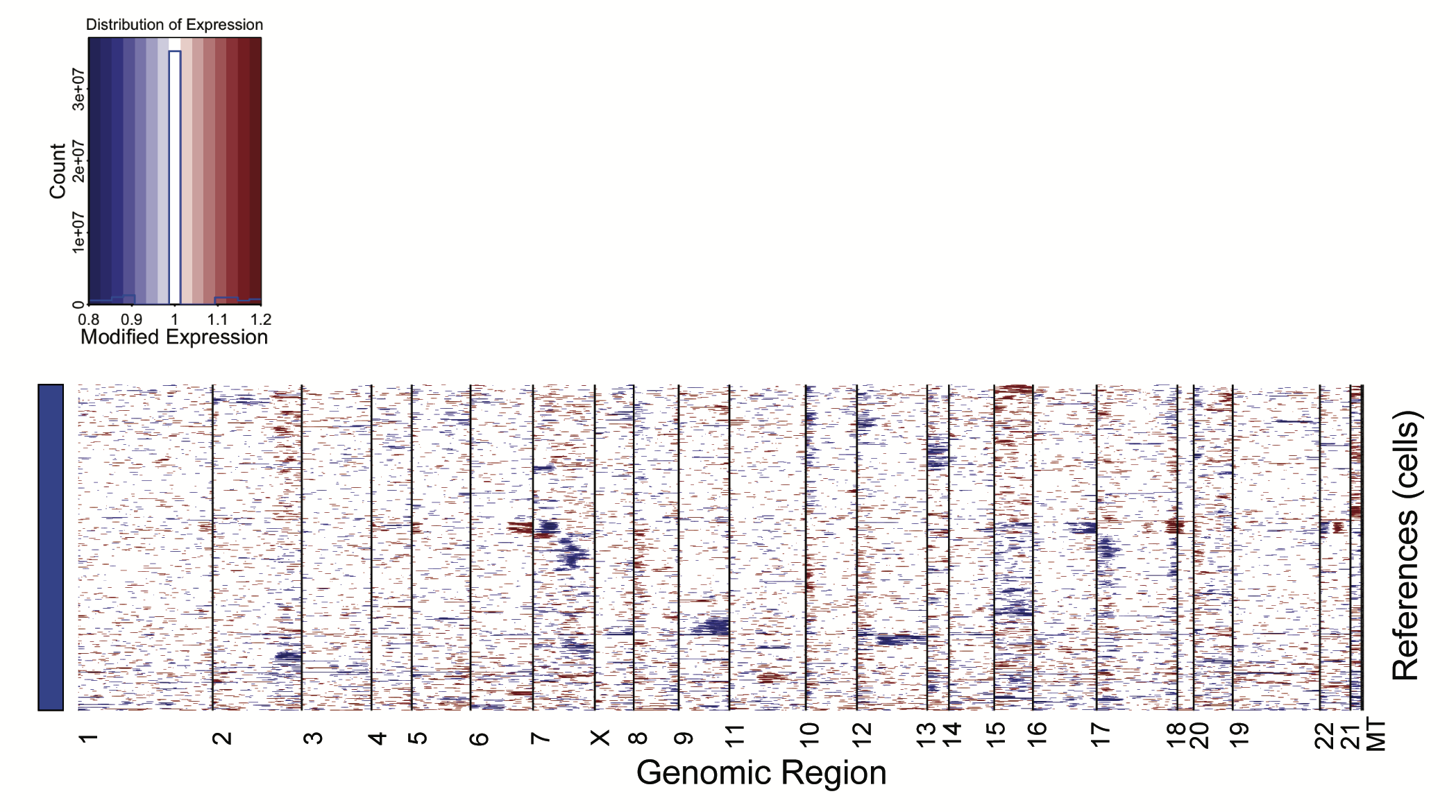

Supplement: S10 Fig — Values are plotted as a heatmap. All PC9-VU sublines were compared against this baseline in the CNV analysis. The data underlying this figure can be found in github.com/QuLab-VU/GES_2021. CNV, copy number variant. (TIF) [file pbio.3000797.s011.tif]

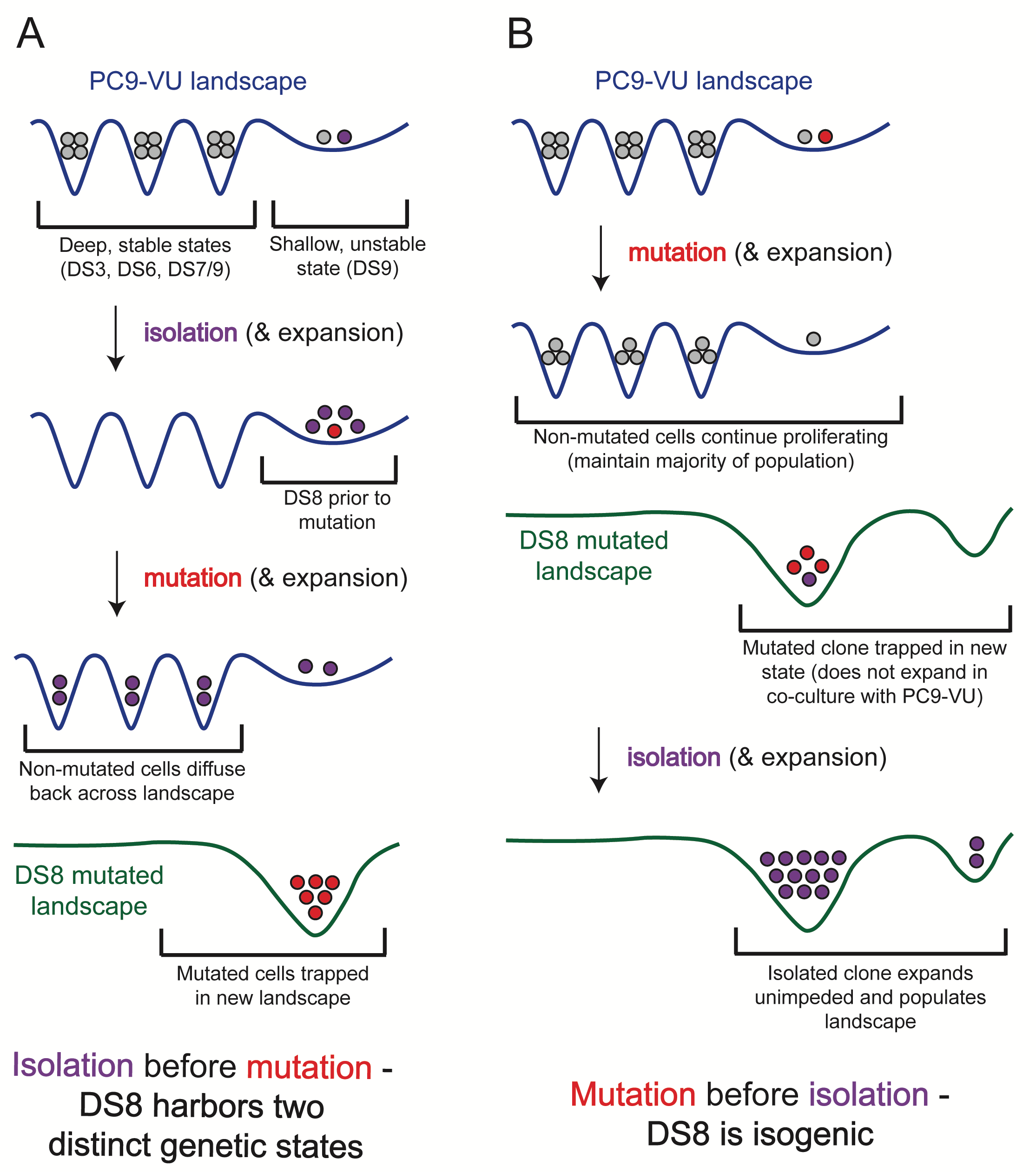

Supplement: S11 Fig — (A) Multiple genetic states hypothesis. In this scenario, a genetic resistance mutation was acquired after the DS8 subline was established. Assuming the mutant state does not outgrow the original genetic state (i.e., a “selective sweep”), both genetic states should coexist within the subline. (B) Single genetic state hypothesis. In this scenario, a genetic resistance mutation emerged within the PC9-VU parental population and a cell containing that mutation was isolated to establish the DS8 subline. To explain our single-cell transcriptomics data, we hypothesize that cell–cell interactions between mutant and PC9-VU cells increase the death rate for mutant cells, making them a small proportion (<2%) of the total PC9-VU population. (TIF) [file pbio.3000797.s012.tif]

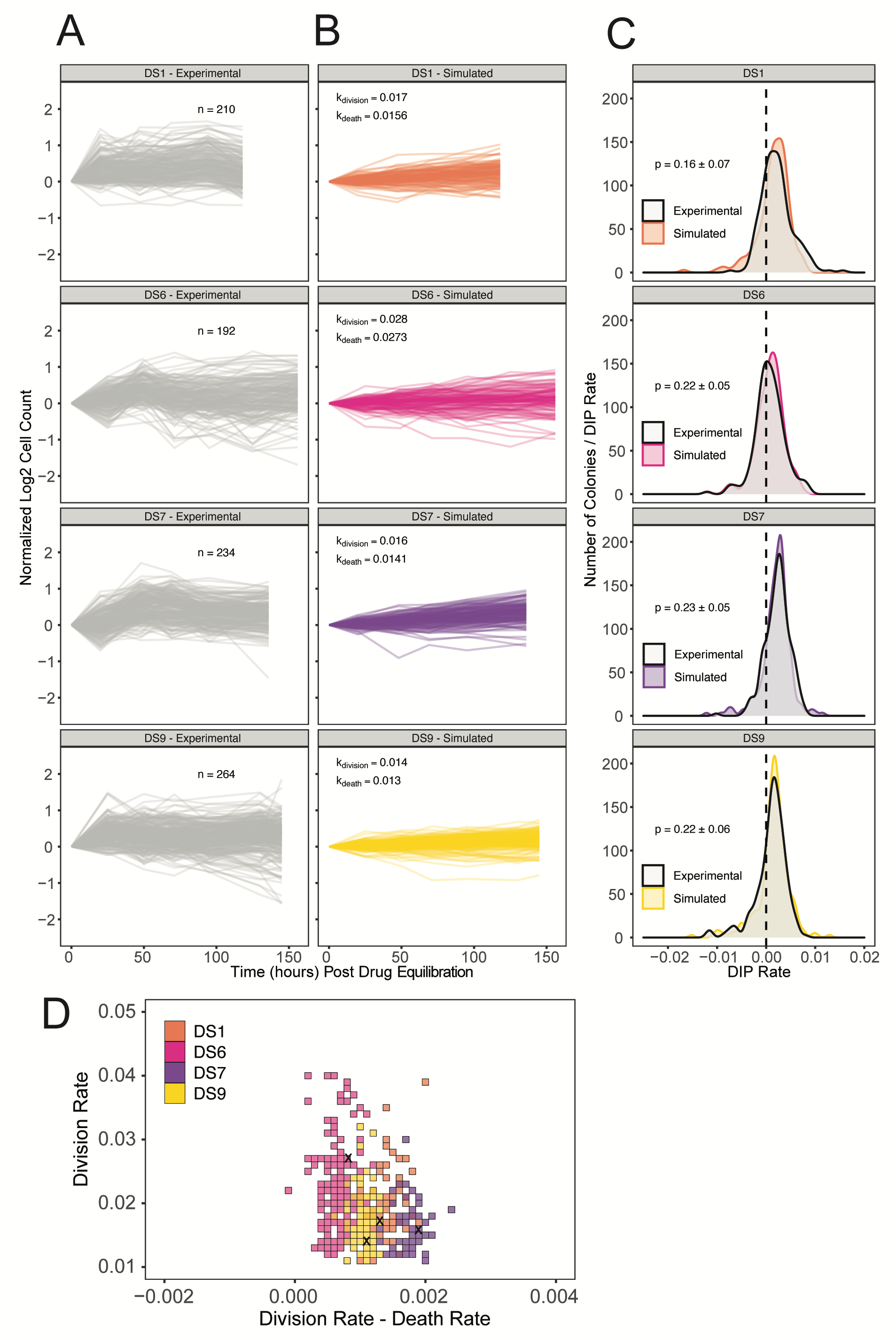

Supplement: S12 Fig — (A) Experimental cFP time courses for 4 PC9-VU sublines (DS1, DS6, DS7, and DS9) in response to 3 μM erlotinib (same data used to generate DIP rate distributions in Fig 2D of the main text). Each trace corresponds to a single colony, normalized to 72 h postdrug treatment. Only colonies with cell counts greater than 50 at the time of treatment were kept; n represents the number of colony traces for each subline. (B) Simulated cFP time courses generated using division and death rate constants that closely reproduce the experimental time courses in A. Trajectories are normalized to the time at which the simulated drug treatment was initiated and simulated cell counts are plotted only at experimental time points. Although the same number of simulations were initiated as the number of colonies (n) in the corresponding experiment (see panel A), only simulated colonies with cell counts >50 at the time of simulated drug treatment are shown. (C) Comparison of experimental and simulated DIP rate distributions calculated from time courses in A and B. Distributions are compared statistically using the AD test (see Materials and methods). Bootstrapped p-values are shown (mean and standard deviation). Dashed black line signifies zero DIP rate, for visual orientation. (D) Parameter scan of division and death rate constants for the 4 sublines in A–C. For each pair of rate constants, we ran model simulations (same number as corresponding subline), calculated DIP rates, compiled them into a distribution, and then statistically compared against the corresponding experimental DIP rate distribution using the AD test (bootstrapped). All p < 0.05 are colored white, indicating lack of statistical correspondence to experiment. × denotes a division and death rate constant used in B. The data underlying this figure can be found in github.com/QuLab-VU/GES_2021. AD, Anderson–Darling; cFP, clonal fractional proliferation; DIP, drug-induced proliferation. (TIF) [file pbio.3000797.s013.tif]

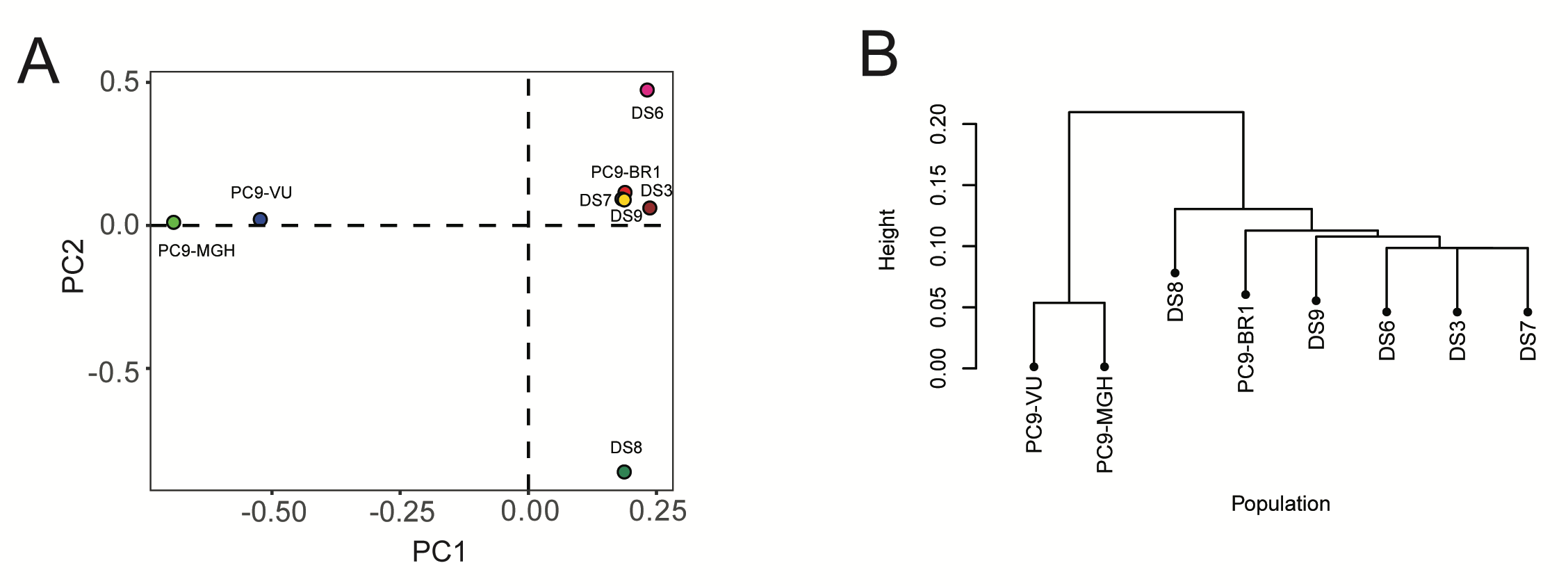

Supplement: S13 Fig — (A) PCA of PC9 genotypes. Using a subset of SNPs in approximate linkage equilibrium, a genetic covariance matrix was calculated. The covariance matrix was converted to a correlation matrix to achieve appropriate scaling and PCA was run to identify SNP eigenvectors (loadings of the principal components). PC9 cell line family members are plotted along the principal component axes. (B) Hierarchical clustering of PC9 genotypes. Using an identity-by-state analysis, a matrix of genome-wide pairwise identities was calculated. Hierarchical clustering was performed on these identities to determine sample relatedness. The data underlying this figure can be found in github.com/QuLab-VU/GES_2021. PCA, principal component analysis; SNP, single nucleotide polymorphism. (TIF) [file pbio.3000797.s014.tif]

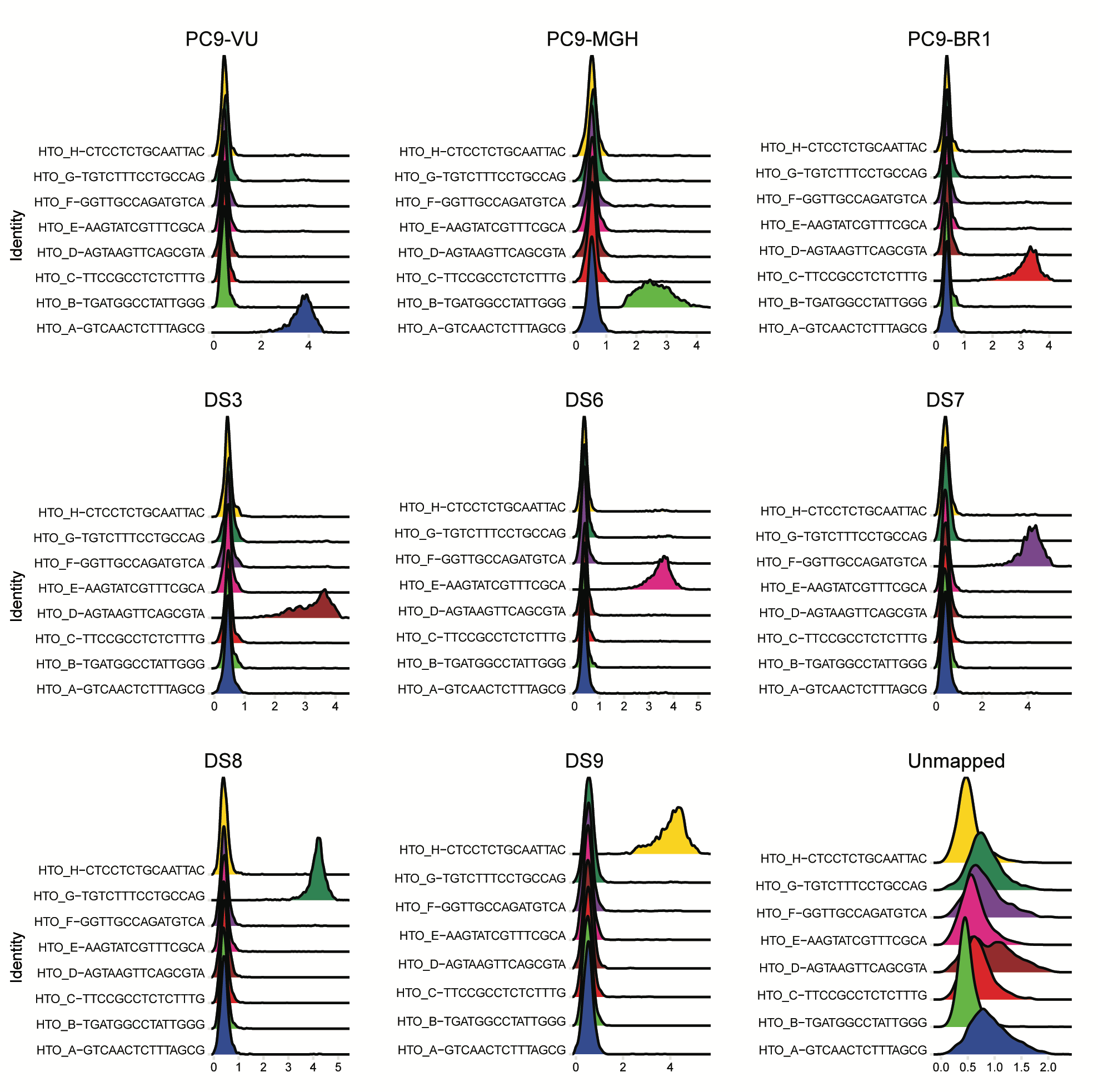

Supplement: S14 Fig — Proportional representation of cell populations with each of 8 specific “hashtag” antibodies, based on the HTO expression level. Each sample has a single corresponding HTO, while a minority of the HTO reads were unmapped. The data underlying this figure can be found in github.com/QuLab-VU/GES_2021. HTO, hashtag oligonucleotide. (TIF) [file pbio.3000797.s015.tif]

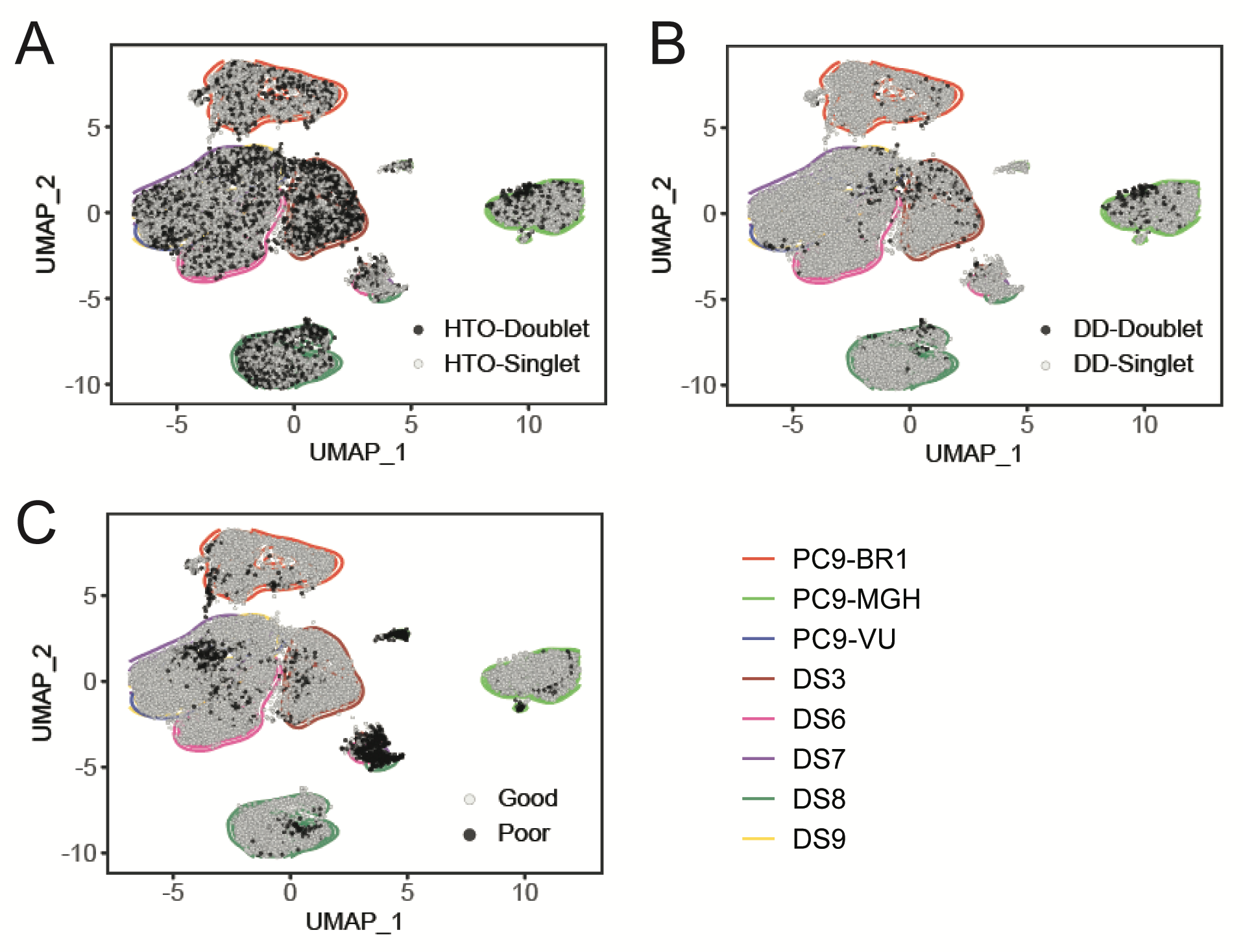

Supplement: S15 Fig — (A) Cell hashing allowed for detection of cell multiplets (1 droplet with more than 1 cell) because multiple HTOs would be detected for a single cell barcode (i.e., droplet). Cells were segregated into singlets and doublets (i.e., multiplets). All detected cell transcriptomes were visualized using UMAP, noting singlets and doublets. (B) Automated DD was performed on the detected cell transcriptomes. Doublets were predicted and noted on the same UMAP visualization as in A. (C) Cells were scored based on number of features (e.g., genes) and count of detected RNA molecules. Cells with scores below a specified threshold (see Materials and methods) were classified as “poor” quality. Quality of each cell was noted on the same UMAP visualizations as in A and B. A total of 7,892 cells passed singlet and quality control thresholding. The data underlying this figure can be found in github.com/QuLab-VU/GES_2021. DD, doublet detection; HTO, hashtag oligonucleotide; scRNA-seq, single-cell RNA sequencing; UMAP, Uniform Manifold Approximation and Projection. (TIF) [file pbio.3000797.s016.tif]
