# Supplementary material for: An in vitro model of tumor heterogeneity resolves genetic, epigenetic, and stochastic sources of cell state variability
Source: PLoS Biol. 2021 Jun 1;19(6):e3000797. doi: 10.1371/journal.pbio.3000797 (PMC8195356; doi:10.1371/journal.pbio.3000797)
Supplement: Attachment — Submitted filename: ReviewerResponses.pdf [file pbio.3000797.s017.pdf]

RESPONSES TO REVIEWER COMMENTS

Reviewer #1:

Overall comments:

In their manuscript, Hayford and coworkers apply a framework relating genetics, epigenetics, and stochasticity to derivatives of the non-small-cell lung cancer line, PC9. The framework is Waddington's epigenetic landscape, where the tent strings underneath the landscape are pulled to varying extents depending on mutations or other perturbations (PMID 29590606). Cell-to-cell heterogeneity rocks the landscape and opens the possibility of a cell escaping one attractor and entering another. The authors deeply describe the molecular and phenotypic characteristics of PC9 subclones along with polyclonal lines from different laboratories, performing exome sequencing, RNA-seq at the population and single-cell level, and fractional proliferation assays. The goal is to reconcile mutations, single-cell fluctuations in gene-expression state, and drug-induced proliferation rates by using the framework. It is difficult to say whether they accomplished that goal, but the multi-omics data provide a lot of observations that could be followed up on in the future.

The major issue I have with the manuscript is bloat. There are three types of experiments here—1) exome sequencing, 2) single-cell/bulk transcriptomics, 3) time-lapse recordings of inhibitor-treated cells—and associated analyses. Given the scope of the work and the findings, 87 pages is not impressive but agonizing. The Waddingtonian landscape does not require two pages to introduce, nor do we need a dozen examples of how noise can arise in biology. The experimental results in Figure 6 are a carbon copy of Figure 4 expanded by one PC9 subclone, artificially creating a density to the work that it does not deserve. The Supplementary Note is not a note at all but instead another dozen pages of text that editorializes on the model, gives an introductory primer on the different types of mutational events, and speculates on the role of stochasticity. The only text that really belongs there is the description of the growth model, as it is essential to understand the results shown in the main text. The authors should go back and look critically at every text passage, supplementary figure subpanel, and yet-another analysis toward justifying why each element is absolutely essential for their message.

Response: We appreciate the Reviewer's insightful comments here and below. To address the concern about bloat, we have made significant changes to the manuscript, including streamlining the Results section, combining all subline omics data into a single figure (revised FIG. 4), and removing large portions of the Supplementary Text. Specifically, in the main text, we moved the descriptions of the different levels of heterogeneity (genetic, epigenetic, stochastic) from the Results section to the Supplementary Text, removed the subsection on DS8 and integrated its text into that for the subline omics data and the model simulations, and shortened the Discussion section. In the Supplementary Text, we have removed multiple sections, including those on the different types of mutations, the computational model (all relevant information about the model is now included in Methods), and the role of stochasticity in the epigenetic landscape. The revised Supplementary Text has only four

sections: the descriptions of the three levels of heterogeneity, a discussion on the relationship between transcriptomics and epigenetics, an expanded discussion of the rationale behind the genetic-to-epigenetic analyses that we perform (including semantic similarity), and a presentation of two hypotheses about the origin and genetic composition of the DS8 subline. We feel that these four sections convey important information necessary for understanding our results and conclusions in the main text. We have also significantly reduced the number of definitions in Supplementary Table S1 and removed the GO term matrix from Supplementary Table S2 and replaced it with a list of gene names from FIGS. 3D and 4D. Please note that we did have to increase the number of Supplementary Figures from 13 to 17 to address a variety of concerns by all three Reviewers.

Quite possibly, there are some interesting results here. If the UMAPs of Fig. 3G, 4G, and 6C are reassembled into a single graph, one sees an outlier transcriptional state in PC9-VU, which is recapitulated almost exactly in the DS9 clone—could this clone be thought of as a cancer stem cell-like founder for PC9s, with an epigenetic landscape that is shallow (or noisy) enough to populate the other basins? Reciprocally, the DS8 clone appears to be trapped in the outlier state—does the increased mutational burden mechanistically explain the altered landscape? Lip service to a mutated ABC transporter and a silent mutation in RELN is insufficient. Real biological connections that are plausibly mechanistic would allow the authors to look for related connections in other NSCLC datasets.

<u>Response</u>: The Reviewer raises a very interesting question regarding the small number (~2%) of PC9-VU and DS9 cells that fall in the same region of transcriptomic space as DS8. Since our genomics data suggest that DS8 is genetically distinct from PC9-VU, one possible explanation is that the outlier cells constitute a small subpopulation of genetically distinct DS8-like cells within the PC9-VU and DS9 populations. However, DS9 was single-cell cloned, just as DS8 was. Therefore, for this to be true, a DS9 cell would have had to acquire the same genetic mutations as DS8 and the outlier cells in PC9-VU *after* the DS9 subline was established (otherwise, we would expect DS9 to look like DS8), which seems unlikely. A second possibility is the one the Reviewer puts forth, that the outlier cells reside in a shallow basin within the PC9-VU epigenetic landscape. This seems plausible but there are some complications with this theory as well. For example, DS7 and DS9 cells overlap almost entirely within the transcriptomic space but we do not find any DS7 (or any other subline) cells in the outlier state. Also, as the Reviewer points out, DS8 cells appear to be trapped in this state. The most straightforward explanation for this is that genetic mutations altered the landscape for DS8, stabilizing the outlier state and either eliminating or making inaccessible (by increasing the quasi-potential energy barrier) the PC9-VU states. However, for this to be true, the genetic mutations in DS8 would have had to be acquired after the DS8 subline was established. Otherwise, there would be genetically distinct DS8-like cells in the parental population, which leads to the same problem discussed above. But if that is the case, why do we not observe any DS8 cells in the PC9-VU region of transcriptomic space? Could it be that DS8-like cells *do* exist within the PC9-VU parental line but their growth is limited by interactions with the other PC9-VU phenotypes?

As the discussion above illustrates, the origin and nature of the outlier cells in PC9-VU and DS9 and their relationship to DS8 is a compelling but, as of yet, unsolved problem. Our best guess at present is that the outlier cells in PC9-VU and DS9 do reside in a shallow basin within the PC9-VU epigenetic landscape, mutations in DS8 stabilize this state and were acquired *before* the DS8 subline was established, and cell-cell interactions between DS8-like cells and other PC9-VU phenotypes maintain the population of these cells at low levels in the PC9-VU parental population. We discuss these possibilities in some depth in the Supplementary Text ("*Multiple possible routes to a new genetic state in DS8*") and provide schematic illustrations in Supplementary FIG. S14. Of course, this is just informed speculation at this point. Providing a molecular explanation is outside the scope of the current work but is something we definitely plan to pursue in the future. Please also see our response to Major Point #5 below.

Major points:

1. Novelty and utility of the genetic-epigenetic-stochastic (G/E/S) framework. The manuscript pseudo-presents a set of ideas at the beginning as if they have never been described before. In fact, prior work by the authors follows almost the exact same organizing principles, with "genotype" substituted for "drug treatment" (PMID 29590606). It is fine to build off past examples—simply cite the best and most-informative examples and get on with the new results here. Second, while I do not doubt that the G/E/S framework provides a straightforward physics-based analogy, I question whether it provides more than a cartooned explanation of the findings. The framework has no predictive value, and the sketches are so flexible that it appears to be consistent with whatever data are at hand. Is there anything that could falsify the G/E/S framework? A theory that explains everything explains nothing.

<u>**Response**</u>**: We thank the reviewer for this comment and apologize for any confusion that our presentation of the G/E/S framework may have caused. We certainly do not claim that these ideas are novel to us, nor that they have never been described before. To the contrary, we explicitly stated at the beginning of the third paragraph of the original manuscript that this three-tiered view of tumor heterogeneity has been proposed previously and provided numerous citations to relevant works. We followed that with a statement, which we believe to be true, that these ideas have not been fully accepted within the cancer research community because of a lack of strong experimental evidence to support them. In making these statements, we were attempting to frame the purpose of our paper as providing such evidence. Indeed, as the Reviewer notes, we used the same theoretical framework in our previous publication (PMID: 29590606) but did not include molecular or genomic profiling. This work builds on that prior work, albeit in a different cancer type. We recognize, however, that by focusing so heavily on the framework we failed to convey this message. Therefore, we have made significant changes to the manuscript to reduce the focus on the G/E/S framework (a term we no longer use) and instead focus on the experimental data that we collected and the model simulations we performed. For example, we modified the title to remove any reference to the framework. Also, we now present the framework briefly in the second paragraph of the Introduction, refer the reader to the Supplementary Text for additional details, and move immediately into the Results. We return to the framework in the second paragraph of the**

Discussion, where we use it to interpret our results and draw conclusions. We follow that with a paragraph where we briefly review some recent papers that we believe are consistent with the framework. We then conclude the manuscript with a paragraph where we use the framework to muse on possible reasons for why many modern cancer therapies fail and hypothesize on strategies that may prove more effective. We hope these changes more clearly communicate the goals of our manuscript and convey the proper deference to the wide body of literature on which our work is based.

With regard to the predictive value of the framework, we do believe that it can produce testable hypotheses and be falsified. A simple example of this are the transcriptomic profiles of the sublines. The theory asserts that single-cell cloning will produce genetically and epigenetically homogenous populations, at least for some period of time (depending on the depths of the basins). Therefore, one prediction would be that the transcriptomic profiles of the PC9-VU sublines will overlap with different portions of the PC9-VU parental transcriptome, which we indeed see. If we did not observe this in our data for the majority of the sublines, that would call into question the validity of the theory. Furthermore, the DS8 subline is an obvious exception. Since its transcriptomic profile is so distinct from that of the PC9-VU parental line, one would predict that it is genetically distinct, which we argue is true based on our data. If this were not the case, it would again call into question the validity of the theory. A third example relates to the outlier state in PC9-VU and DS9 that resides in the same region of transcriptomic space as DS8 (also see our response to Major Point #5 below). The nature of this state is unclear at present but in the Supplementary Text and Supplementary FIG. S14 we propose two hypotheses based on the G/E/S framework that could be tested experimentally. Moreover, as the Reviewer also notes (Major Point #5), this state could be similar to the pre-resistant state reported by Shaffer et al. (PMID: 28607484). An important question, however, is whether cells linger in that state or simply transition in and out randomly due to stochastic noise. The G/E/S framework would predict that cells linger since the state is a basin in an epigenetic landscape. In principle, this prediction could be tested by experimentally measuring residence times in the state and comparing against timescales associated with random fluctuations.

We hope that our responses above are convincing enough to assuage the Reviewer's concerns regarding the usefulness and legitimacy of the G/E/S framework as a theoretical concept. We also refer the Reviewer to our response to a similar question below by Reviewer #2 (Major Concern #2).

2. Lack of clarity on the meaning of "Unknown" exonic mutations. Amidst all the text in the Supplementary Note defining SNPs, indels, and missense mutations, I can find no explanation for how exome sequencing can yield an "Unknown" mutation. The only way I can fathom is if the capture beads pulled down collateral bits of intronic and extragenic sequence, but then those mutations should be omitted from the data reporting as they are not in exons. Most of the mutations reported in Fig. 3D and S10D fall into the "Unknown" category, so it is critical to know what these are.

<u>Response</u>: We apologize for the lack of clarity. “Unknown” corresponds to a category of mutations detected by the dNdScv tool that are not single-nucleotide variations. The unknown mutations have been annotated as the different types of mutations (primarily inframe and frameshift insertions/deletions). A small number of complex substitutions were also noted as Unknown mutations. With the addition of copy number variation data (revised FIGS. 3E and 4E; see response to Major Point #4 below), we have removed the complex mutations from the dataset. These changes have been updated in the figures (revised FIGS. 3D and 4D).

3. Lack of consistency in exome sequencing results. There are also inconsistencies in the exome data that were not acknowledged. For example, ZNF717 is reported to be mutated in three of the four clones in Fig. 4D. One would expect that frequency of mutation to be picked up in the PC9-VU bulk population, but no ZNF717 mutation is reported in Fig. 3D. Furthermore, only two ZNF717 mutations are reported among the five clones shown in Fig. S10B, which is supposed to be a one-clone expansion of the data in Fig. 4D.

<u>Response</u>: The inconsistencies noted by the reviewer were due to running the dNdScv tool on different subsets of the data. When running the tool on the entire 8 sample cohort together, most of those inconsistencies were resolved. Based on a preliminary check, depending on the samples input into the algorithm, certain samples pass or fail a specified confidence threshold (dNdS = number of nonsynonymous divided by synonymous mutations in a specific gene across all input samples). In the case of specific mutations (such as ZNF717), samples were close to the threshold. With all 8 samples together, a few genes (MUC3A, GOLGA6L7, MADCAM1, GOLGA8F, CHD4, GOLGA6L4, IGFN1) still exhibited mutations across multiple samples. These mutations were in different locations in the gene and, therefore, considered different mutational events. For clarity, those events were removed from the visualization so that only unique mutated genes (instead of distinct events) are included. Improved visualizations can be seen in FIGS. 3D and 4D.

Furthermore, frequencies of mutations can (and probably were) muddled by the heterogeneity of each cell population. Cell line versions, like PC9-VU, likely had more genomic variability than the more homogeneous single-cell derived sublines. Due to the thresholds needed for variant detection, this variability would actually result in fewer detected mutations in a heterogeneous population because the confidence needed to detect these mutations would not be acheived. However, in a homogeneous population, mutations likely exist in all of the cells and, therefore, would have more statistical confidence. This result is present in the total number of detected variants (Supplementary FIG. S4A) as well as the large overlap between PC9-VU and its derived clones (Supplementary FIG. S4E). However, as mentioned above, running the dNdScv tool with all 8 samples combined has resolved this inconsistency.

4. Copy number alterations are ignored. The experimental implementation of the G/E/S framework focuses entirely on mutations and does not consider the impact of chromosome stability and copy-number alterations. In a larger, thematically-similar study of HeLa cells (PMID 30778230), copy-number alterations were a driving force for heterogeneity among derivatives of the line. The authors could address this concern without any further experiments by applying

inferCNV to their bulk RNA-seq data on each PC9 derivative and clone. CCLE RNA-seq data on PC9s could serve as a "normal" starting reference, assuming that CCLE obtained their PC9s directly from the original source.

<u>Response</u>: We thank the reviewer for this suggestion. In the revised manuscript, we now use inferCNV on our single-cell RNA-seq data to predict potential copy number variations. Those results are now shown in revised FIGS. 3E and 4E. Specifically, cell line versions (revised FIG. 3E) were run with no reference set (such as CCLE data, to avoid potential batch effect errors), while sublines (revised FIG. 4E) were run with PC9-VU as the reference (revised Supplementary FIG. S11). PC9-MGH seems to have the clearest changes in the cell line version cohort, with amplifications in chromosomes 6, 11, and 22 and a deletion in chromosomes 16 and 19. In the subline cohort, DS8 has major amplifications in chromosomes 6, 18, and 22 and major deletions in chromosomes 7 and 13. These results are now discussed in the second and third subsections of "Results" in the main text.

5. Equating transcriptional and epigenetic states. The authors acknowledge that epigenetics is more conventionally viewed in terms of histone and DNA modifications. My issue is less with the measurement type and more with the implied lifetime of the state if it is framed epigenetically. For a state to be epigenetic, there should be some degree of heritability before the state "relaxes" over several generations. But, some transcriptional states just randomly switch because of noise or other reasons that have nothing to do with epigenetics (see https://www.biorxiv.org/content/10.1101/379016v2 for a nice description of the distinction between the two). The outlier state in PC9-VU and DS9 would be a prime candidate for assessing lifetime, if there were surface markers that could be sorted for (PMID: 28607484).

<u>Response</u>: We agree with the reviewer on this point and have tried in the Supplementary Text ("*Genetic, epigenetic, and stochastic sources of tumor heterogeneity*" – formerly in the main text) to emphasize heritability and characteristic timescale as ways to differentiate epigenetic processes from stochastic processes. We generally think of the different sublines as corresponding to different epigenetic states and the spread of cells within the transcriptomic spaces of the sublines as being reflective of stochastic variability. However, the outlier state in PC9-VU and DS9 that resides in the same region of transcriptomic space as DS8 is admittedly a bit of a mystery at this point. Our intuition is that it corresponds to a shallow basin within the PC9-VU epigenetic landscape, i.e., it is less stable than the other states but more than simply the result of stochastic fluctuations. It is possible that a cell in this state acquired a genetic mutation that stabilized the state, resulting in what we see in DS8. We have added some language to this effect at the end of the third paragraph of the Discussion, where we cite the two works mentioned above by the Reviewer. We have also modified Supplementary FIG. S14 to illustrate these concepts visually. Of course, we cannot know whether our intuition is correct until we assess the lifetime of the state experimentally, as the Reviewer suggests. Unfortunately, with our current data, there are not enough DS9/PC9-VU cells (~2%) that fall within the DS8-like state to differentiate them from other cells in their respective groups, making biomarker identification infeasible. However, we plan to pursue this question further in the future. We are currently working on a follow-up study in melanoma on phenotypic state

transitions that is similar in scope and which we hope to expand to PC9 and other EGFR-mutant NSCLC cell lines shortly.

6. Proof-by-intimidation reporting of the omics data. Whereas the cFP and DIP experiments are generally clear, well described, and informative, the omics data is presented in a manner that overwhelms the reader more than it empowers them. Taking Fig. 3D-E as a case in point, the text is illegible at anything approaching a normal page, the shading that redundantly encodes the bar graph to the right is imperceptible even on a retina display, and the cancer genes list is effectively devoid of information (while being crammed with gridded lines and microscopic font). I would recommend a phased presentation, where the most-critical results are presented in main figures, and the gory details are presented ONCE in the supplementary figures (i.e., Figure S10A-D and removing Fig. 4A, D-F).

<u>Response</u>: We apologize for the manner in which we presented our omics data in the original manuscript. It was certainly not our intent to intimidate the reader nor overwhelm them. To address this issue, we now present the omics data over two main figures (revised FIGS. 3 and 4), the first for the cell line versions and the second for the sublines (including DS8), and numerous Supplementary figures (revised Supplementary FIGS. S2A, S4—S12, S15—S17). As suggested, we present what we feel are the most critical results in the main figures and include in the Supplementary figures only those data necessary for supporting these results. In the main figures, we first present the whole exome sequencing data by stepping through from the total number of mutations (FIGS. 3A/4A) to the unique mutations (FIGS. 3B/4B), the number of IMPACT mutations (FIGS. 3C/4C), the annotated gene lists predicted by dNdScv and colored by mutation type (FIGS. 3D/4D), and the copy number variations (FIGS. 3E/4E). We have removed the gene names from revised FIGS. 3D/4D and put them in Supplementary Table S2 to avoid the problem of them being crammed and too small to read. We have also moved the cancer-associated gene mutation heatmaps and mutation classification pie charts, which were in the original main figures, to Supplementary FIG. S5. We follow this by presenting the UMAP projections of the single-cell transcriptomics data (FIG. 3F/4F) and, finally, the GO term analyses comparing variations in the genome to differential expression at the mRNA level (FIG. 3G/4G). Results from our revised semantic similarity analysis are now presented in Supplementary FIG. S10. We hope that this reorganization of the figures sufficiently addresses the Reviewer's concerns.

7. Excluding trivial clone-to-clone differences. As understood, the PC9-VU clones were isolated from a polyclonal population of PC9-VU cells ectopically expressing H2B-mCherry. The abundance of the H2B marker could readily change the epigenetic state of cells, as mCherry is much larger than H2B, and there are many unpublished anecdotes of H2B-mCherry cells behaving oddly, possibly the result of artifactually opening chromatin that should normally be closed. At the minimum, the authors should assess the relative abundance of H2B-mCherry in the different clones and confirm that the overall expression of the label is not dramatically different among clones.

<u>Response</u>: We thank the reviewer for this suggestion. PC9-VU clones were isolated from a polyclonal population of PC9-VU cells expressing H2B-RFP. Prior work from our lab (PMID: 22886092, PMID: 27135974) has shown no discernible effect of the RFP molecule on drug-response phenotypes. To directly address the Reviewer's concern, we provide bulk RNA-seq normalized expression data among H2B gene family members across all eight samples in the study (see below). We see minimal variation in H2B expression level across these samples. Please note that we have not included this data in the manuscript in an attempt to limit length and number of figures as much as possible.

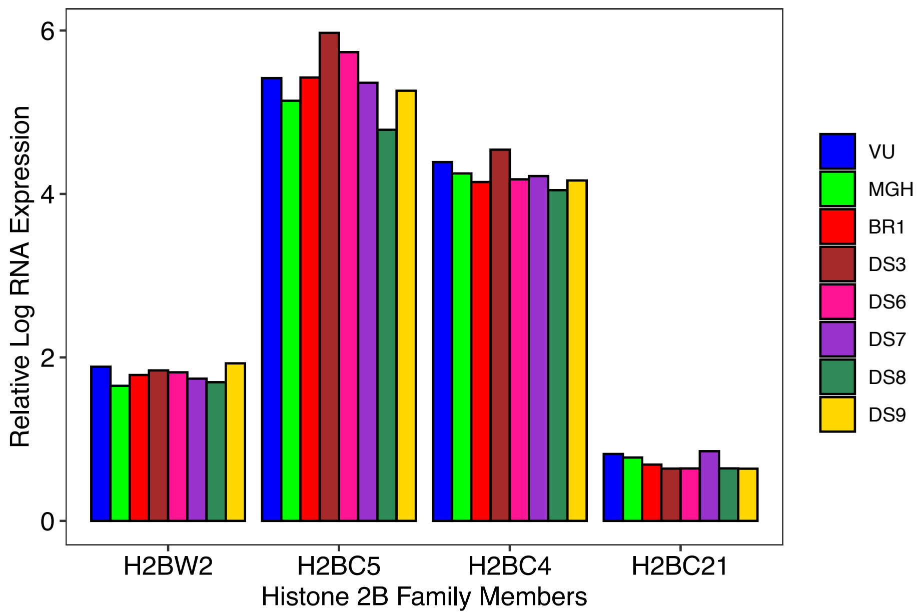

Minor points:

1. The "semantic similarity" is possibly a good way to bring together the mutational data with the transcriptomic data. However, there must be some type of interval estimate on the score and the polyclonal baseline used for comparison. Stability of the scores could be estimated by cross validation.

<u>Response</u>: We agree with the Reviewer on this point. To address this issue, we have modified the method for calculating the semantic similarity score to generate a 95% confidence interval as well as a baseline. In Supplementary FIG. S10 of the revised manuscript, we present these scores for each cell line version and subline normalized relative to their baselines (i.e., scores > 0 are considered significant). Note that we have moved the semantic similarity analysis into the Supplementary Figures because the analysis is somewhat more nuanced and less clear given these changes. We have replaced this analysis in the main text with a more straightforward correlation analysis of shared GO terms between data modalities (IMPACT mutations and differentially expressed genes).

We also refer the Reviewer to our response to Reviewer #2 (Major Concern #5), where we provide a more detailed explanation of the modifications that we have made to the calculation of the semantic similarity score as well as our rationale for moving the results of the analysis to the Supplementary Figures.

2. I can understand the motivation for "withholding" the DS8 clone in the presentation, but it really exacerbates the bloat of the manuscript. Also, in the presentation of the first four clones, it is not clear why DS9 and DS3 were selected, as they are very similar in their response to EGFR inhibition. Perhaps it is better to frame those two clones as a "within group" comparison to estimate how different the transcriptional states can be and yet yield similar drug-induced responses.

<u>Response</u>: We thank the reviewer for this comment. By presenting the DS8 subclone near the end of the "Results" section we were attempting to simplify the presentation since our conclusion is that it is genetically distinct from the other subclones. However, we understand that this had the opposite effect and only confused the presentation. To address this, all data duplication of the sublines has been removed from the figures and we now present all omics data from the subclones in FIG. 4 of the revised manuscript and in various Supplementary Figures. We have also integrated the section on DS8 from the original manuscript into the subsections in the revised manuscript on subline heterogeneity and model results (third and fourth subsections of the Supplementary Text, respectively).

With regard to the choice of the four sublines (DS3, DS6, DS7, DS9), we based the decision on their DIP rate distributions rather than their population-level drug responses, which can be deceiving. As mentioned at the beginning of "One PC9-VU subline is genetically distinct, while all others are transcriptomically distinct from each other" in the revised manuscript, DS3 has a peak in the negative DIP rate range, DS6 has a peak near zero, and DS7 and DS9 have very similar distributions in the positive DIP rate range. If we had the chance to redo our selections, we may have decided to choose to DS4 instead of one of DS7 or DS9. However, we do not believe that this would have affected the primary conclusions of this work. Importantly, we were limited to four (of six) of these sublines because, together with DS8 and the three cell line versions, this was the maximum number of samples we could run on a single 10x Genomics single-cell RNA-seq experiment (using cell hashing) and comfortably get more than 1000 cells per sample. This allowed us to avoid batch effects that could have complicated our analyses.

3. The formulation of the parameter inference of the stochastic DIP models is generally well done. However, I am not clear on why the division rates appear to flat-line at a division rate of ~0.045. I hope that was not simply the maximum division rate tested. Also, it is inappropriate to use large p values as a metric of goodness of fit. Much better would be to report the K-S statistic and shade to indicate the minimum value(s) that are deemed of interest.

<u>Response</u>: We apologize for not including the full range of parameter pairs in our original parameter analysis. In the revised manuscript, we have rectified this by including division rate constants up to 0.08 h^{-1} (revised FIGS. 5D,H and Supplementary FIG. S13D). With regard to p-

values, we no longer shade tiles in these figures based on p-value. We simply define a p-value cutoff and shade all tiles that exceed the threshold a color corresponding to the subline. Please note that based on a suggestion by Reviewer #2 (see Minor Comment #1) we now use the Anderson-Darling (A-D) test rather than the Kolmogorov-Smirnov test. This involved bootstrapping the experimental DIP rate distribution (100 times) and comparing each to the simulated DIP rate distribution to generate a distribution of p-values. If the <p> - sdev(p) > 0.05, we considered the comparison to be significant.

4. This is not entirely the fault of the authors, but I find the V3 output of the GeneRanger software annoying for GEO uploads. The barcodes file precludes reviewers from quickly spot-checking the raw data against the figures. If the gene names could be exported along with the unique barcodes, that would be preferred.

<u>Response</u>: We will submit a request to GEO to add the full matrix (with row and column names) to the database before publication of our manuscript.

Typographical points:

1. Page 5: The reference to Harris et al. should only refer to 89.

<u>Response</u>: We thank the reviewer for pointing out this error. We have replaced "Harris et al." with "we" since we are referring to two papers from our group: Tyson et al. (2012) *Nature Methods* (PMID: 22886092) and Harris et al. (2016) *Nature Methods* (PMID: 27135974).

2. Page 7: The statement "The result above illustrate…" is inaccurate. The only thing that can be said is that the mutations are associated with transcriptional differences.

<u>Response</u>: We apologize for the confusion. The full quote that the Reviewer is referring to is "The results above illustrate that genetic differences among cell line versions translate to epigenetic differences at the transcriptomic level." In this work, we are defining "epigenetic" to mean any heritable state of a cell that is not associated with the DNA. As discussed in the revised Supplementary Text ("Relationship between transcriptomics and epigenetics"), the transcriptomic state is just one lens through which the epigenetic state can be viewed. However, to avoid confusion, we have removed the sentence that the Reviewer is referring to from the main text and rewritten the paragraph (page 6 of the revised manuscript) discussing the quantification of the connection between genomic and transcriptomic states in the cell line versions.

3. Page 11: The statement "However, individual basins are not discernable." is false. The outlier basin is clearly visible in Fig. 3G.

<u>Response</u>: We apologize for the confusion. In this sentence, we were specifically referring to the scRNA-seq data for the PC9-VU cell line version shown in FIG. 3F of the main text. We were trying to make the point that it was not obvious from that data that there were discrete states

"hidden" within it but obscured by stochastic fluctuations. This was meant to set up the statements in the following sentences about how the scRNA-seq data for the different sublines overlap with different portions of the PC9-VU parental transcriptomic space (FIG. 4F of the main text), which we view as evidence that PC9-VU has an associated epigenetic landscape with multiple phenotypic attractors (FIGS. 1 and 6 of the main text). We maintain this argument in the revised manuscript but have removed the sentence in question to avoid confusion.

4. Figure S8: There is no need for a legend here. Just label the data points.

<u>Response</u>: **As suggested, in Supplementary FIG. S8 we have removed the legend and labeled the data points by sample.**

5. Supplemental Note, Page 12. "reduce" should be "reduced".

<u>Response</u>: **We thank the Reviewer for pointing this out. The Supplementary Text has been completely rewritten in the revised manuscript. The sentence that the Reviewer is referring to is no longer present.**

Reviewer #2:
[identifies himself as Aaron S Meyer]

Overall Comments:

Hayford et al. provide a framework to deconvolve drug response variability into genetic, epigenetic, and stochastic sources in an in vitro heterogeneous tumor model. They explore the genetic and epigenetic differences that explain drug response variability using mutational impact, single-cell differential gene expression, and semantic similarity of gene ontology analysis across 3 versions of the PC9 NSCLC cell line and 8 sublines of PC9-VU. The authors conclude that the cell line versions, as well as single cell-derived sublines differ at genetic and transcriptomic levels. They argue that their framework could be employed to account for all levels of heterogeneity when evaluating cancer treatment.

Overall, the manuscript presents a comprehensive analysis of heterogeneity within a cell line model. It presents a valuable analysis that would be of interest to the drug response and tumor heterogeneity communities. Before it is suitable for publication, however, there are some major concerns outlined below that must be resolved. I expect the authors can fully address these concerns with text changes and some additional computational analysis.

<u>Response</u>: **We thank Dr. Meyer for these overall comments. We address each aspect pointwise below.**

Major concerns:

1. I appreciate that the authors have aimed to be comprehensive in their definitions and discussion. However, parts of the paper seem very wordy. As one example, genetic, epigenetic, and stochastic heterogeneity are defined within separate sections, and then again defined within a supplementary table. If there are suitable references the paper can use to explain these definitions, this could help to focus the paper on the new results presented here. It would also make the manuscript more accessible overall.

Response: We thank Dr. Meyer for this comment. In the revised manuscript, we have moved the descriptions of the three levels of heterogeneity (genetic, epigenetic, and stochastic) to the Supplementary Text ("Genetic, epigenetic, and stochastic sources of tumor heterogeneity") and have reduced the number and lengths of the definitions in Supplementary Table S1. We believe the manuscript is now much more streamlined and accessible.

2. I understand the comparison to the ideas proposed by Waddington but think this needs (1) be clarified, and (2) its ultimate usefulness highlighted. The Waddington landscape model specifically describes a developmental process, and how barriers between states might increase as development progresses. Where is the analogous process here? In what way can one use the attractor model as a testable hypothesis, or is this a tautological idea?

Response: We appreciate Dr. Meyer's comments and welcome the opportunity to clarify our thoughts on these important issues. We acknowledge that Waddington's landscape was developed as a conceptual framework for understanding cellular differentiation during development. However, we do not believe that the framework is exclusive to that setting. Rather, we view development, or more specifically, a developmental hierarchy as a certain type of Waddington landscape comprising successive basins of decreasing quasi-potential energy. As cells traverse down this hierarchy, from stem cell to progenitor cell and so on, reverse transitions, or "de-differentiation", become increasingly unlikely. This explains why barriers between states increase as development progresses. In cancer, we believe the same processes occur, except without a well-defined hierarchy. This is why tumors are composed of multiple phenotypes, some of which are "stem-like", the degree of stemness being a function of how deep or shallow a basin is within the landscape. This perspective has been discussed by many others, including Marusyk et al. (2012) (PMID: 22513401). We have added some language to the end of the second paragraph of the Introduction to address the issue of Waddington's landscape in development and how it applies in cancer.

With regard to the usefulness of the Waddington landscape concept in cancer, we argue that it can be extremely valuable in deciphering and interpreting complex and sometimes contradictory experimental results. For example, in a recent publication of ours (PMID: 29590606), we observed melanoma cell populations that when treated with a BRAF inhibitor expanded, regressed, expanded again, and then entered a state of balanced division and death that we termed "idling". At first, we had a hard time interpreting this behavior. However, once we started thinking in terms of epigenetic landscapes and built a simple model similar to that

used in this work except with transitions between states, the explanation became clear: the drug changed the landscape, knocking the population out of equilibrium; the complex dynamics we observed were the result of a re-equilibration of the population over the new, drug-modified landscape. We also believe that the Waddington landscape concept can explain why therapies based on targeting specific genetic alterations or rare cell types, i.e., cancer stem cells (CSCs), so often fail. In the first case, since each genetic state has an associated epigenetic landscape with multiple phenotypes that cells diversify across, some of these phenotypes may be suited, by their molecular makeup, to survive drug treatments targeting that genetic state. These surviving cells may then acquire genetic resistance mutations, leading to tumor recurrence and metastasis. In the second case, a CSC can be thought of as a shallow basin within an epigenetic landscape. Killing cells in that basin does not eliminate the basin. Thus, once the drug is removed, cells from "adjacent" basins can transition and repopulate the basin, restarting tumor growth. We discuss these issues and more in the final paragraph of the Discussion section.

Finally, we do believe that the Waddington landscape model can produce testable hypotheses. For example, if the PC9-VU subline transcriptomes did not overlap with different portions of the PC9-VU parental transcriptome, that would have severely damaged our hypothesis that the sublines constitute individual basins within a common PC9-VU epigenetic landscape. Moreover, the fact that the DS8 subline transcriptome does not overlap with that of PC9-VU led to the hypothesis that it is genetically distinct from PC9-VU, which was verified by our whole-exome data. Another example that has yet to be tested relates to whether the genetic mutation within DS8 was acquired before or after the subline was established. In the Supplementary Text ("*Multiple possible routes to a new genetic state in DS8*") and Supplementary FIG. S14, we present two possible scenarios based on the concept of the Waddington landscape. These hypotheses could be tested through some combination of single-cell cloning of DS8, scRNA-seq, and DNA barcoding to track lineages within co-cultured populations. We are currently devising experiments along these lines.

We also refer Dr. Meyer to our response to a similar question above by Reviewer #1 (Major Point #1).

3. The authors must at least acknowledge that heterogeneity can arise from environmental and pharmacologic differences. There is extensive literature on the contribution of these factors, even within simple tissue culture systems.

<u>Response</u>: We agree with Dr. Meyer and thank him for this comment. Environmental and pharmacological factors are actually implicitly accounted for in the genetic-epigenetic-stochastic framework that we advocate for in this manuscript. Environmental factors can be seen as inputs or boundary conditions to the molecular networks that underlie the epigenetic landscape. Therefore, similar to the effect of genetic mutations (mentioned briefly in the Introduction), changes in the environment can alter the nature of the landscape, changing the number, types, and abundance of phenotypic states within a tumor. This is true for microenvironmental differences between primary and metastatic tumors as well as macroscale

factors that act on the level of the patient, such as pollution or diet. Fluctuations in these environmental factors can also drive phenotypic state transitions, in the same way that intrinsic fluctuations in gene expression do.

Pharmacological factors have a similar effect but since they are usually small-molecule drugs, we think of them as new molecular species within the network, rather than inputs or boundary conditions. Adding new molecular species to a network can obviously change the nature of the epigenetic landscape. This concept is actually the basis for a recent publication from our lab (PMID: 29590606), where we measured the response of BRAF-mutant melanoma cell populations to a BRAF inhibitor over time. We used a three-state population dynamics model similar to the one used in this work, except we allowed phenotypic transitions between states. Our conclusion from that study was that the BRAF inhibitor changed the topography of the epigenetic landscape and that the complex short-term dynamics that we observed at the cell population level were due to a re-equilibration of cells across the drug-modified landscape.

To address Dr. Meyer's concern, we have added sentences to the first and second paragraphs of the Introduction listing out various sources of non-genetic heterogeneity and how changes in environmental and pharmacological factors can alter epigenetic landscapes within a tumor. We hope this, together with the explanations above, sufficiently address this issue.

4. The tense throughout the manuscript is inconsistent which leads to confusion. The authors seem to start by referring to their experiments in the past, and observations in the present, which reads well. However, this changes a few times throughout the text.

<u>Response</u>: We apologize for this oversight. We have revised the manuscript to use consistent tense throughout, with experiments referred to in past tense and observations in present tense, as suggested.

5. Semantic similarity is an interesting idea for demonstrating conserved molecular programs. Can the semantic similarity score be evaluated compared to a meaningful null model for significance? For example, randomized gene sets of the same size from those genes expressed in PC9? Maybe it can also be shown that the similarity between types of molecular measurements is higher within a cell line (e.g. BR8) as compared to between lines? I am concerned that GO enrichment might simply be a reflection of the genes expressed in this cell line, and that semantic similarity simply scales with the number of impacted genes.

<u>Response</u>: We would like to thank Dr. Meyer for this comment. In response to this comment and those from the other Reviewers, we have performed an exhaustive investigation into the semantic similarity metric and discovered that it does appear to scale (at least partially) with the number of impacted genes (see plot below). Since the score averages the *maximum values* from each column and row of the GO semantic similarity matrix to arrive at a single value, adding more GO terms (from more genes) acts to increase the average. Put simply, if we have a list of values *x*, the maximum value of *x* can only increase as more values are added to the list, it can never decrease. Thus, semantic similarity scores for samples and modalities with

more genes tend to skew higher than those with fewer genes. To address this issue, we have modified the implementation of the metric in the following ways:

1. ***Developed a confidence interval metric* – Instead of choosing GO terms to be input into the semantic similarity calculation based on a p-value threshold ($p<0.05$), as in the original method), GO terms are now selected if $r < 1-p$, where r is a random number between 0 and 1. This creates longer GO term lists, which are then fed into the semantic similarity calculation. But instead of creating a "max average", as in the original version, we take the largest 1000 scores from the similarity matrix and calculate a median and 95% confidence interval.**

2. ***Created a baseline score with confidence intervals* – This score is created by compiling, for each sample (cell line version or subline), a random list of genes (from the hg38 reference) with the same number of terms as those for each data modality. These random gene lists are run through the semantic similarity pipeline to generate a baseline median score and confidence interval (as described in point *1* above). The baseline median value + one standard deviation is subtracted from the experimental semantic similarity score to produce a normalized value. Thus, semantic similarity scores > 0 are considered significant.**

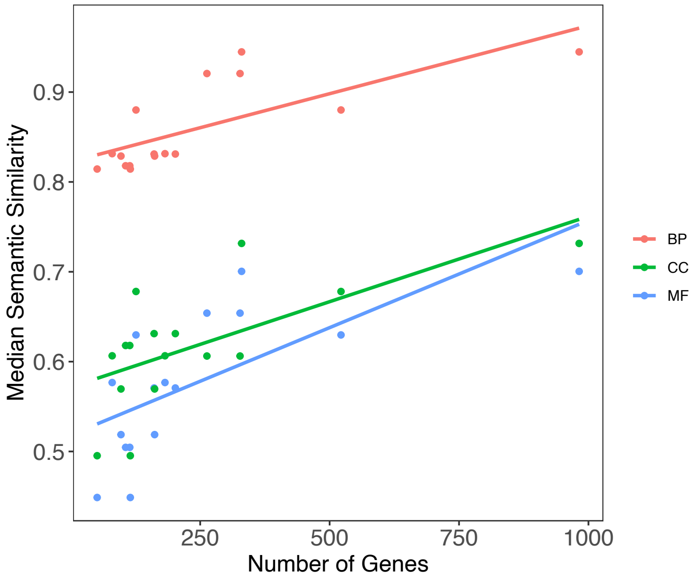

Furthermore, there is also a question as to how much confidence to assign to the different GO term categories: "Biological Process" (BP), "Molecular Function" (MF), and "Cellular Component" (CC). The categories have drastically different numbers of terms in the GO network structure (BP ~14000 >> MF ~6000 > CC ~2000). We have taken the position here that the number of terms is a reflection of the "biological knowledge" contained within each category. Thus, we put more stock in the BP semantic similarity score than we do in that for MF and more in MF than for CC. This makes the interpretation of the semantic similarity analysis more nuanced. Because of this, we decided to move the results of this analysis to Supplementary FIG. S10.

In the main text, in place of the semantic similarity analysis, we have added correlation plots for shared GO terms across modalities. We performed a GO over-enrichment analysis on each gene list (as in the first part of the semantic similarity calculation; see above) and identified terms that are significantly over enriched ($p<0.05$) for both modalities (IMPACT mutations and differentially expressed genes). We then plotted these shared terms on axes of log p-values, along with correlation coefficients if there were enough shared terms to warrant it (revised FIGS. 3G and 4G). We saw that, generally speaking, cell line versions (other than PC9-VU) and DS8 have positive correlation coefficients, while the other sublines have negative coefficients or not enough terms to calculate a correlation. Together with the semantic similarity analysis, we feel these results support our position that the cell line versions and the DS8 subline are genetically distinct but the other PC9-VU sublines are not. We have modified the main text to make this point.

6. The author's conclusions from their SSA model are overreaching and inconsistent. I agree that they can conclude their DIP rate measurements are consistent with the SSA model but do not agree that they can say much more. It is unclear to what extent their measurements are powered to reveal other forms of heterogeneity if present. Second, they are using one measure of phenotypic heterogeneity, when "epigenetic" heterogeneity is a much more encompassing term (e.g. drug response to other compounds). Third, their parameter uncertainty is simply that, and not evidence that the cells themselves fluctuate.

Response: We would like to thank Dr. Meyer for this comment and apologize for any confusion that our discussion of the SSA results may have caused. As Dr. Meyer points out, the most important conclusion of these results is that the DIP rate distributions for all of the PC9-VU sublines (other than DS8) can be described by a model with a single cell state. This provides strong evidence that the sublines are monoclonal (revised FIG. 5), which is consistent with the idea that they constitute basins within an epigenetic landscape (revised FIGS. 1 and 6). We have modified the main text to emphasize this point exclusively.

Other conclusions that we could draw from these results are admittedly more subtle and we have removed them from the manuscript to avoid confusion. Conceptually, we were attempting to connect DIP rate distributions to intracellular fluctuations in molecular species concentrations. Since intracellular signaling networks govern cell fate decisions, it follows that fluctuations in the concentrations of species involved in cell fate decision making would result in randomness in division and death events and, hence, distributions of DIP rates. However, we are obviously not measuring fluctuations in protein levels to directly tie to cell fate decisions in this work and our main conclusions are not reliant on making this direct connection. Hence, we made the decision to remove these statements from the manuscript.

Also, we apologize for any confusion regarding the terms "phenotypic heterogeneity" and "epigenetic heterogeneity", which we use effectively interchangeably. Much of the material provided in the Supplementary Text (which was originally in the main text) and the definitions in Supplementary Table S1 are attempts to avoid confusion on such issues. Basically, we define

a phenotypic, or epigenetic, state as a basin within an epigenetic landscape. Thus, a phenotypically, or epigenetically, heterogeneous population is one in which cells occupy numerous such basins. Examples are the cell line versions (VU, MGH, BR1); counterexamples are the monoclonal PC9-VU sublines (DS3, DS6, DS7, DS9). Critically, the epigenetic landscape is defined in terms of a set of intracellular molecular species, i.e., the molecular "state space". Which species define the landscape depends on context, e.g., in our case, the context is sensitivity to the EGFR inhibitor erlotinib, so the state space is technically defined by all molecular species involved in cellular response to erlotinib. However, this means that the phenotypic, or epigenetic, states can be different in different drugs. DS3 and DS6, for example, which are distinguishable in erlotinib (both by DIP rate and transcriptomic state) may not be under a different drug; conversely, DS7 and DS9, which cannot be distinguished in erlotinib, may be distinguishable under a different drug. Thus, while we agree with Dr. Meyer's statement that epigenetic heterogeneity can broadly refer to drug response to multiple compounds, we are using it in a narrower sense in our manuscript to mean degree of dispersion across multiple epigenetic basins within a specific context. We hope this explanation is sufficient to alleviate any concerns regarding this issue.

Minor comments:

1. When comparing the SSA model to DIP measurements, the Anderson-Darling test should be more sensitive to differences as compared to the Kolmogorov-Smirnov test. Also, bootstrap resampling is necessary to prevent bias in the test statistic. This is summarized well here: https://asaip.psu.edu/articles/beware-the-kolmogorov-smirnov-test/.

Response: We thank Dr. Meyer for this comment and online resource. In the revised manuscript, we have changed the statistical test to the Anderson-Darling test and bootstrap resampled the data (100 times) to compare to each simulated result, creating a range of p-values. We chose $\langle p \rangle - \mathrm{sdev}(p) > 0.05$ as the condition under which we cannot reject the null hypothesis that the experimental and simulated distributions are the same. In addition, we have updated revised FIG. D,H and Supplementary FIG. S13D to remove shading based on p-value and simply color the tiles if this condition is met (see also response to Reviewer #1, Minor Point #3).

2. I would not call PC9 a prototypical or archetypal example of lung cancer. It is a commonly used model, but like any cell line model (or any one tumor) presents a limited view of all lung cancer.

Response: We agree with this point and have replaced "archetypal" with "commonly used" in the revised manuscript.

3. There are a couple of places where there is the note: "(see below)". It is not clear what the authors are referring to.

Response: We apologize for the confusion. We have modified the text in the manuscript to refer to other sections by name, rather than simply "below" or "above".

4. In figure 2B, the y-axis is density—is the magnitude of this quantity meaningful (e.g. in units of cell number)? If not, it would be better to normalize the distributions such that the y-axis would be 0-1.

<u>Response</u>: Density in FIG. 2B,D is in units of [# of colonies / DIP rate], so that the areas under the curves equal 1. We did this to facilitate direct comparisons among the distributions since each is based on a different number of cFP colonies. To avoid confusion, we have changed the y-axis labels in these plots to "Number of Colonies / DIP Rate".

5. In figure 3, it is not clear to which plot "CV=12.84" belongs. Same in figure 4.

<u>Response</u>: We apologize for the confusion. The CV values correspond to panels (A) in Figs. 3 and 4. We have modified the figures by moving the values into the middle of the circular bar plots to avoid confusion.

<u>Reviewer #3:</u>

Overall Comments:

Hayford et al tackle the issue of disentangling the genetic, epigenetic and stochastic sources of heterogeneity in cancer response to treatment. The authors tackle this problem with a combined approach of theoretical and mathematical modeling with genomics, single cell transcriptomic and experiments on drug response in three cancer cell lines and several monoclonal sublines derived from one of these three lines. This is a very important problem which has relevant repercussions on drug design and development of therapeutic strategies in cancer. The system and the approach used are clever and effective and the results are interesting. However, I think the authors could have done a better job in analyzing the large amount of data they produced, in particular the transcriptomic data, which could provide a much more detailed and informative characterization of the epigenetic landscape of the system. Moreover, the manuscript is massive, highly redundant and difficult to read because the information is poorly organized and scattered through main text, methods and supplementary note (the pdf is also missing line numbers which makes it difficult to review). Most importantly, the results can be presented in a much more concise and effective way. As the most striking example, almost all the data reported in figure 4 is repeated in figure 6 and supplementary figure S10 (even with different color codes), with the addition of another subline, making figure 4 B C D E F G H and I panels completely redundant. Also, in another example, the authors separate the sublines in different subplots in figure 2C when Figure S4 shows that sublines can be put in the same plot which is more concise and makes it easier to compare between them. A profound reorganization of the figures and of the results section is necessary before publication.

<u>Response</u>: We would like to thank the Reviewer for the insightful critique provided here and in the comments below. We have attempted to address each concern to the best of our ability and believe our revised manuscript is much stronger as a result. To address the concerns raised in this comment, we have removed and streamlined portions of the main text and Supplement. Specifically, we have moved the descriptions of the three levels of heterogeneity (genetic, epigenetic, stochastic) to the Supplementary Text, made more concise arguments in the Introduction, shortened the Glossary of Terms in Supplementary Table S1, shortened the Discussion section, removed the section in the Supplementary Text pertaining to types of genetic alterations, and included line numbers in both the main text and Supplement. We also strove to mention key information only once in the revised text, including consolidating all explanations of methods used in the Methods section. For the figures, we have consolidated all subline DIP rate distributions into a single plot overlaid with the PC9-VU parental distribution (revised FIG. 2D) and placed scRNA-seq data for all sublines, including DS8, into one UMAP projection together with contour lines for the cell line versions to facilitate comparison (revised FIG. 4F). We have also added analyses that provide insight into the biological basis for UMAP dimensions and clusters, performed a copy number variation analysis, and modified the semantic similarity analysis. Additional details are provided in our responses to the critiques below.

Major points:

1. Single cell RNA seq data is a tremendous resource to characterize the state of a cell but here we are left with very little information; the authors should extract more interpretable information from their data: For example, why don't they start from UMAP including all their data like the one they have in supplementary fig S7 i.e. both lines and sublines instead of splitting their analysis into three in the main text? Further, UMAP is a powerful visualization technique but it also lacks interpretability without integration or further analyses to characterize what the UMAP dimension 1 and 2 represent biologically. What are the molecular signatures and biological processes that separate the different clusters in the UMAP? What can we learn from these signatures about the epigenetic landscape? Which basin(s) of attraction - or cell state(s) is (are) i.e. associated with drug resistance? The authors could for example use the following tool to improve interpretability of their analysis: https://github.com/YosefLab/VISION.

<u>Response</u>: We thank the Reviewer for this comment. To address the first concern regarding the scRNA-seq data being split into three parts, in the revised manuscript we have combined all of the subline data, including for DS8, into a common UMAP plot (revised FIG. 4F). We still present the scRNA-seq data for the cell line versions separately (revised FIG. 3F) to coincide with the presentation in the main text and to avoid overlap in the UMAP plot between the sublines and PC9-VU parental cell line, which would make the two sets of data hard to distinguish. However, we have included topographical contour lines for the cell line versions in the UMAP plot for the sublines in order to facilitate direct comparisons (revised FIG. 4F).

Regarding biological interpretability of the UMAP dimensions and clusters, we used the VISION tool recommended by the Reviewer to generate distributions for 50 Hallmark gene signatures,

taken from the MSigDB database (gsea-msigdb.org/gsea/msigdb/collections.jsp), for all cell line versions and sublines (Supplementary FIG. S9). In short, we see broad hallmark processes that differentiate the populations and, therefore, the UMAP axes. For example, the KRAS signaling score is enriched in PC9-MGH and the PI3K/AKT/MTOR signaling score is enriched in the PC9-VU cohort. Since PC9-MGH and PC9-VU differ along the UMAP_1 but not UMAP_2 axis (revised FIG. 3F), this suggests that UMAP_1 has some relation to signaling through the parallel PI3K/AKT/MTOR and RAS/RAF/MEK/ERK pathways. Furthermore, PC9-BR1 is enriched for cholesterol homeostasis but not for Hedgehog signaling, while DS8 is enriched for DNA repair and unfolded protein response but not for the p53 pathway. PC9-BR1 and DS8 differ along the UMAP_2 but not UMAP_1 axis (revised FIG. 4F), suggesting that these processes are relevant for the interpretation of the UMAP_2 axis. We have included a summary of these results in TABLE 1 of the revised manuscript and briefly discuss them in the Results section.

2. The GO semantic similarity analysis between the genetic and epigenetic level is potentially interesting but we lack information about the results and interpretation of this analysis. For example, the authors provide just an example of the analysis for subline DS8 in supplementary table S2 but they only provide the GO identifiers and not the names making any attempt to understand and interpret the analysis very hard. What are the biological processes and molecular functions that drive the semantic similarities between the genetic and epigenetic level of the cell lines? Are those processes or functions shared among some of the lines or sublines?

Response: To address the concern about biological interpretation, we have included in revised FIGS. 3G and 4G correlation plots for the GO terms that have shared significance between the data modalities (IMPACT mutations and differentially expressed genes). We display on the plots the GO term labels for all terms with $-\log_{10}(p) > 2$ for either modality, to give some insight into the molecular programs active within each sample. Please note that these correlation plots replace the semantic similarity scores included in the original manuscript. The semantic similarity metric has been modified in the revised manuscript and the analyses moved to Supplementary FIG. S10 (see our response to Reviewer #2—Major Point #5 for further details). We have also removed from Supplementary Table S2 the example GO similarity matrix for DS8 that the Reviewer references in their comment.

We would like to clarify that the goal of the semantic similarity analysis is to determine whether genetic variants within a cell line or subline can explain differential expression at the transcriptomic level within the same cell line or subline. If so, then there is evidence that the samples being compared (i.e., cell line versions, sublines) are genetically distinct. If not, then there is evidence that the samples represent basins within a common epigenetic landscape. Importantly, since we are specifically looking at mutations that are unique to each sample and genes that are differentially expressed within a sample relative to others within the sample group, we would not expect to see many, if any, shared processes. Indeed, there is only one shared term between PC9-VU and PC9-MGH, one between PC9-VU and DS3, and one between DS3 and DS9. No other pairs of samples showed any overlap in their GO terms.

3. In addition, if I understand correctly, the authors only compare each subline against the other sublines both at the genetic and RNA seq level, but wouldn't it be interesting to compare them also against the line from which they are derived?

<u>Response</u>: To address this concern, we have added a mutational overlap between PC9-VU and the sublines (Supplementary FIG. S4E), which shows that the largest degree of overlap, by far, is for all the sublines and PC9-VU. This is unsurprising since the sublines were derived from the PC9-VU parental population. We reference this in the revised text at the end of "Methods: Derivation of single-cell sublines". At the transcriptomic level, we have included contour lines for the cell line versions in the UMAP projection for the scRNA-seq data for the sublines (revised FIG. 4F). This facilitates a direct comparison between the PC9-VU subline transcriptomes and the PC9-VU parental transcriptome, which show significant overlap. We also show a bulk RNA-seq comparison of the PC9-VU sublines and parental in Supplementary FIG. S8A. We hope these inclusions sufficiently address the Reviewers' concerns.

4. Why haven't the authors performed single cell RNA seq of DS1?

<u>Response</u>: As mentioned in our response to Reviewer #1 (Minor Point #2), we had space limitations in the scRNA-seq experiment to incorporate cell hashing and minimize batch effects. With the three cell line versions (VU, MGH, BR1) and the DS8 subline (which was an obvious choice), we had space for four additional samples out of the six remaining sublines. We chose these more or less randomly to cover a range of drug responses, based primarily on their DIP rate distributions (revised FIG. 2D). We chose DS3 because it has a peak in the negative DIP rate range, DS6 because it has a peak near zero, and DS7 and DS9 because they have peaks in the positive DIP rate range. DS1 and DS4 just happened to be the two sublines we excluded. We do not believe that this affected the primary conclusions of our work in any way.

5. It would be nice if the authors could confidently conclude that the BR1 line and the subline DS8, which are both resistant to the drug, achieve resistance through two distinct genetic and/or epigenetic cell states. However, from figure 4 G, (or figure 6 C) it seems to me that a minority of the DS8 cells occupy the same epigenetic state of BR1. It is therefore difficult to conclude that the resistance in DS8 is not driven by those cells that are close to BR1, also considering that the bimodal distribution in the DIP rate would suggest that it is a minority of cells that drive the resistance. Maybe the authors could sort and profile mutations and RNA of the high DIP rate and low DIP rate cells separately and look at where those cells map in the genetic and epigenetic landscape?

<u>Response</u>: We thank the reviewer for this comment. After a thorough re-analysis of the scRNA-seq data, including doublet detection (Supplementary FIG. S17A,B) and stricter thresholds for removing low-quality cells (Supplementary FIG. S17C) , we no longer find overlap between PC9-BR1 and DS8 (revised FIGS. 3F and 4F). Moreover, the addition of copy number variant data (FIGS. 3E and 4E), which shows different amplifications and deletions within the two populations, strongly suggests that PC9-BR1 and DS8 are genetically distinct. Also, we agree

with the Reviewer that sorting high and low DIP rate cells within DS8 and comparing their genomic and transcriptomic signatures is an excellent idea. We plan to pursue this in future work. However, we do not believe that this affects our primary conclusion in this work, which is that DS8 harbors a genetic resistance state that emerged spontaneously from the PC9-VU population.

6. As explained above, authors should try to present the analyses of all lines and sublines combined. It doesn't make sense to repeat the analysis and presentation of the sublines data before and after adding the DS8, it's too redundant. Then, they can easily discuss the different nature of the DS8 with respect to the other sublines referring to the same plots.

Response: We appreciate and agree with the Reviewer's comment. Originally, we were concerned that including DS8, which we conclude is genetically distinct from the other sublines, together with the sublines that we argue are genetically indistinguishable from each other and the parental PC9-VU cell line would complicate the story and confuse the reader. However, we realize that waiting to present DS8 at the end actually caused more confusion than it prevented. Thus, in the revised manuscript, we have consolidated all of the subline scRNA-seq data, including for DS8, together in FIG. 4 of the main text. We have kept the cell line version data separate in FIG. 3, however, to avoid overlap with the subline data and to coincide with how the data is presented in the text.

Minor points:

Main text:

1. First part of results i.e. the lengthy description of the three levels is actually more suitable for introduction and I'd suggest to shorten it to improve readability, or alternatively stick it to the supplementary note.

Response: We agree with the Reviewer on this point and have moved the descriptions of the three levels of heterogeneity (genetic, epigenetic, stochastic) to the Supplementary Text ("Genetic, epigenetic, and stochastic sources of tumor heterogeneity").

2. At page 7, when describing Fig 3 G i.e. the UMAP: 'we see a clear separation of the features for the cell line version in this space' this is confusing as features has been just used to indicate genes/transcript while describing the feature selection. I guess what the authors meant is: 'we see a clear separation of the cell lines in the UMAP gene expression space' or something on these lines. The same applies to the similar sentence at page 9.

Response: We apologize for the confusion. The Reviewer is correct, we used the word "feature" in these sentences to mean the gene expression pattern in the UMAP space. We have modified the language in the text to clarify this.

3. At page 7: 'However most genetic mutations do not result in an altered expression of that gene'. Which gene?

__Response__: We apologize for the confusion. We were referring to the mutated gene itself and the fact that mutations do not always modify expression levels but may modify, e.g., protein function. We have moved the discussion of this issue to the Supplementary Text ("The genetic-to-epigenetic connection") and reworded the text as follows: "*However, it is well known that a mutation in a gene does not always alter the expression level of that gene. Rather, if gene X encodes for a protein that acts as a transcription factor for gene Y, then a mutation in X could alter the protein structure such that it affects the expression level of Y.*" We hope this sufficiently clarifies what we are trying to convey.

4. Why don't you describe IMPACT score just in the methods the first time you use it instead of just mentioning in the methods and then describe it in the supplementary note? Also check IMPACT is always used in upper case (I have seen at least one instance of lower case impact at page 16)

__Response__: We agree with the reviewer and have modified the manuscript to describe the IMPACT score only in the "Methods" section. Also, please note that capitalized "IMPACT" refers to the designation from the Ensembl Variant Effect Predictor (VEP), the database by which we rank mutations. An IMPACT score quantifies the predicted impact (lower case) of the mutation. To avoid confusion, in the revised manuscript, we use "impactful" to distinguish from the VEP designation "IMPACT".

5. You mention you didn't use the default covariates of dNdScv because they are not available for hg38, but you can't [build] your own covariate? There are lots of possible variables (i.e. chromatin state) that impact mutation rate and possibly confound the analyses.

__Response__: We thank the reviewer for this comment. dNdScv was built for the old hg19 genome and the default covariate built by the developers required extensive effort and was based on multiple data modalities. We did explore the possibility of building our own covariate for the hg38 genome (the newest version of the genome and the one on which all sequencing analyses in this work are based) but there are no suggestions through the dNdScv online support to facilitate building one from scratch. Thus, we attempted instead to "lift over" variants from the hg38 to hg19 genome (using the Picard liftoverVCF and CrossMap tools) and ran these hg19 variants through dNdScv using the aforementioned default covariate. Unfortunately, dNdScv does not handle lifted-over variants very well and outputs an error due to some variants having the wrong reference bases. The development page for dNdScv acknowledges this problem but has not yet taken steps to fix it (https://github.com/im3sanger/dndscv/issues/3).

With these concerns in mind, we decided to keep the current version of the dNdScv analysis of the hg38 variants without using a covariate, i.e., *dNdSloc* (which works like traditional dN/dS implementations) using the number of synonymous mutations in a gene to infer the local mutation rate without exploiting additional information from other genes. Considering that we

are just using the tool to identify mutation type and count the number of mutations that could be significant, rather than identifying specific genes of interest, we do not believe that this affects in any significant way the results of our analyses. We hope this is sufficient to allay the Reviewer's concerns.

6. Cell cycle score: no details are provided on how this was computed.

Response: We apologize for this oversight. We have modified the Methods section ("RNA single-cell transcriptome sequencing: *Data analysis*") to state that cell cycle score was computed using the default implementation in the Seurat package (satijalab.org/seurat/v3.1/cell_cycle_vignette.html). We have also included a UMAP plot annotated with cell cycle scores and topographical overlays for each cell line and subline as Supplementary FIG. S12.

7. Feature selection: why filter out highly expressed genes?

Response: We would like to thank the reviewer for this clarifying question. We utilized the default feature selection provided as part of the Seurat pipeline. In the previous version of the manuscript (which used Seurat 2.3.4), we filtered out highly expressed genes as recommended. In the current version (using Seurat 3.2.2), as with the previous version, we used the "mvp" (mean-variance plot) feature selection method, which calculates the average expression and dispersion for each feature and dispersion z-scores to identify variable features while controlling for the relationship between variability and average expression (summarized from the Seurat manual). The feature selection method controls for the strong relationship between variability and average expression. To handle this problem, we used the Seurat-recommended range of 0.1—8 for mean expression values. This method identified a group of variable features (in this case, 574 genes; Supplementary FIG. S6B) that helped highlight biological signal in downstream analyses (see PMID: 24056876 and biorxiv.org/content/10.1101/460147v1 for more details).

8. K means clustering: is it performed on the whole expression matrix or just on the UMAP projection? Also, why k=3? what happens with K=4? Did you try a silhouette analysis to find the optimal number of clusters?

Response: In the original version of the manuscript, we performed k-means clustering on the UMAP projection for the cell line versions (VU, MGH, BR1) and four of the sublines (DS3, DS6, DS7, DS9) independently. We hypothesized k=3 for the cell line versions because there are three distinct versions and k=3 for the sublines because of the large degree of overlap between DS7 and DS9 in the UMAP space. However, to address the Reviewer's concern, in the revised manuscript we use a method, called *NBClust* (jstatsoft.org/article/view/v061i06), to determine the optimal number of clusters (again in the UMAP space). *NbClust* uses a variety of different techniques (including silhouette analysis) and suggests the number of clusters that is shared among the most techniques. For the cell line versions, as expected, three groups was deemed optimal (Supplementary FIGS. S7A,B). For the sublines, including DS8, we found two distinct

groups (DS8 and DS3/6/7/9; Supplementary FIGS. S7D,E), confirming that DS8 is an outlier among the group.

The results are a bit more ambiguous when we consider the sublines without DS8. In this case, *NbClust* determined four clusters (narrowly over six) to be optimal (see plots below). To understand why requires knowledge of the cell cycle states (Supplementary FIG. S12): *cluster 4* is composed almost entirely, and encapsulates virtually all, of the DS3 cells; *cluster 3* is composed almost entirely of DS6 cells but only those in the G1 and S cell cycle states; *cluster 2* is composed of almost equal amounts of DS7 and DS9 cells but only those in the G1 and S cell cycle states; and *cluster 1* is composed of comparable amounts of DS6, DS7, and DS9 cells that are in the G2/M cell cycle state. Overall, we interpret these results to simply illustrate that there is a great deal of overlap between the transcriptomic states of the four sublines DS3, DS6, DS7, and DS9 that makes it difficult to clearly split them into discrete groups. It is for this reason that we have chosen not to include them in the main text but present them here for the Reviewer's benefit. These results do not affect the conclusion presented in the main text that these four sublines are genetically indistinguishable and likely occupy basins within the PC9-VU epigenetic landscape.

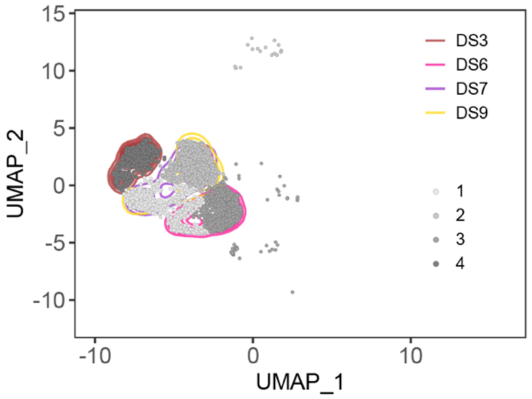

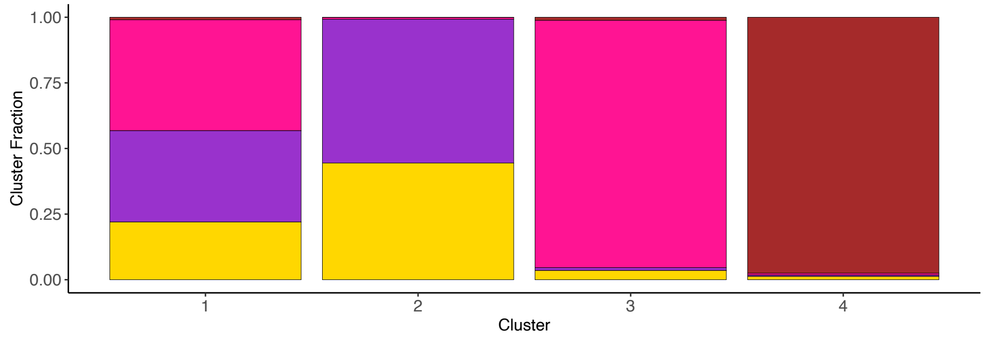

9. In silico modeling. Authors model all sublines with one set of division and death parameters apart from DS8 for which you used two sets. Did they try some sort of model comparison to determine if the sublines are better modeled with a single set or more than one set or they just decided by looking at the distribution of the DIP rate? From the RNA-seq epigenetic landscape it looks like the other lines might also be heterogeneous (i.e. few cells of the DS9 subline occupy the same region of DS8) and so better explained by more than one set of parameters.

<u>Response</u>: To address this concern, we performed a model selection analysis on a two-state model of the DS9 subline, which, as the Reviewer notes, contains a small number of cells in the region of transcriptomic space occupied by DS8. This was a somewhat involved process because it required accounting for two division and two death rate constants and the relative proportions of the two states. To facilitate direct comparisons against the one-state model considered in the main text (Supplementary FIG. S13), we chose the one-state model parameters that best fit the experimental DIP rate distribution (k_{div}=0.015, k_{div}-k_{dth}=0.0012). We held these constant and varied a second pair of division and death rate constants in the ranges k_{div2} = [0.01, 0.08], k_{div2}-k_{dth2} = [-0.0007, 0.0003] and k_{div2}-k_{dth2} = [0.0018, 0.0028], which correspond to regions of the parameter space where the one-state model could not statistically reproduce the experimental DIP rate distribution (Supplementary FIG. S13D). We also varied the proportion of cells in the second state (prior to virtual drug addition) between 1% and 5%. The two-state model thus had five parameters total. We ran a series of *in silico* cFP assays (see Methods) over these ranges of parameter values and initial proportions and calculated DIP rate distributions.

In each case, we also calculated the log-likelihood (LL) given the data,

$$LL = \sum \log_{10} p$$

where p-values were obtained by comparing the experimental and simulated DIP rate distributions at 1000 values of the DIP rate. Specifically, at each value of the DIP rate, we assumed a normal distribution centered on the density from the experimental distribution with a standard deviation equal to 10% of the mean. We then calculated the p-value for the density from the simulated distribution at the same DIP rate within this normal distribution. For model comparison purposes, we calculated the Akaike information criterion corrected for small sample sizes (AICc),

$$AIC_c = 2k - 2\ \ln LL + \frac{2k(k+1)}{n-k-1}$$

where *k* is the number of parameters in the model (two for the one-state model, five for the two-state model) and *n* is the number of experimental data points used to generate the DIP rate distribution. We include a plot below showing the AICc distribution that we obtained from this procedure. Of the 7,810 *in silico* cFP simulations that we performed, zero had a smaller AICc than the one-state model (red dot).

We emphasize that this analysis, which was quite time consuming, was for only *one* of the 55 pairs of one-state division and death rate constants that can reproduce the DS9 experimental DIP rate distribution (Supplementary FIG. S13D) and that DS9 is only one of seven sublines that we consider. Given the heavy computational cost that would be associated with repeating this analysis over all pairs of division and death rate constants for all sublines and the fact that the

analysis presented here confirms that a one-state model is by far the most likely explanation for the observed DS9 DIP rate distribution, we decided not to pursue this direction any further. We have included the description and results of this analysis here for the benefit of the Reviewer but have not included it in the revised manuscript to cut down on length, which was a common criticism of the original manuscript by all Reviewers. We hope this is sufficient to address the Reviewer's concerns regarding this issue.

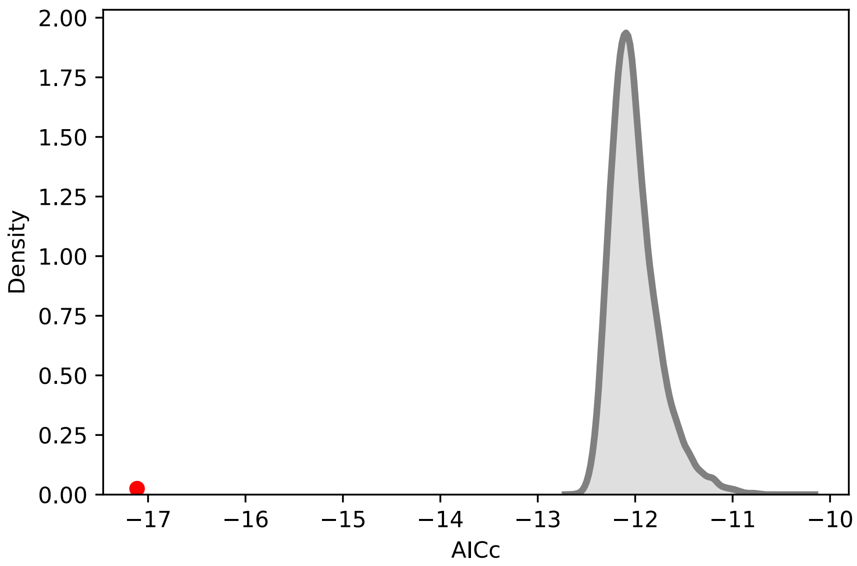

Figures:

1. Figure 2 A and C y axis 'Normalized log 2 cell count'

<u>Response</u>: We have fixed this in the figures.

2. Figure 3 A, Figure 4 A, these circular barplots are less readable than simpler linear ones

<u>Response</u>: We thank the reviewer for the suggestion. We tried to use linear bar plots instead but they were too long to fit well in a figure panel. Thus, with respect, we decided to keep the circular bar plots for the sake of layout and visualization.

3. Figure 3 D, E and Figure 4 D, E Axes and legends are particularly small and hard to read, please uniform them with the ones on the other panels.

<u>Response</u>: We have modified these figures (FIGS. 3D, 4D, and Supplementary FIG. S5 in the revised manuscript) to make the axes labels and legends larger for these panels. Also, for the heatmaps in revised FIGS. 3D and 4D, we have moved the gene names to Supplementary Table S2 to avoid clutter. The gene names are not really important for conveying the main message of the plots, which is that the cell line versions and sublines can be distinguished by gene sets.

Supplementary note:

1. Most of the supplementary note is so long, detailed and describing such basic knowledge (i.e. the description of the different types of mutations etc.) which is more appropriate for an introduction to genetics or for a PhD dissertation rather than for this manuscript.

<u>Response</u>: We agree with the Reviewer and have significantly shortened the Supplementary Text. We now only include four sections describing: 1) the three levels of heterogeneity (genetic, epigenetic, stochastic); 2) the relationship between transcriptomic and epigenetic states; 3) the method that we use to relate genomic variants within a cell line or subline and differential expression at the transcriptomic level; and 4) possible interpretations of our DS8 data. We believe these four topics are important for advancing the storyline of our manuscript but not necessary within the main text.
