# Supplementary material for: An in vitro model of tumor heterogeneity resolves genetic, epigenetic, and stochastic sources of cell state variability
Source: PLoS Biol. 2021 Jun 1;19(6):e3000797. doi: 10.1371/journal.pbio.3000797 (PMC8195356; doi:10.1371/journal.pbio.3000797)
Supplement: Attachment — Submitted filename: ReviewerResponses.pdf [file pbio.3000797.s018.pdf]

<u>RESPONSES TO REVIEWER COMMENTS</u>

<u>Reviewer #1</u>:

Hayford et al. PLoS Biol Manuscript #PBIOLOGY-D-20-01505R2

The revision of Hayford et al. has been considerably streamlined. The fog of readability has mostly lifted, but that has uncovered additional conceptual and technical concerns that must be addressed or clarified.

Major points

1. "Distance" on the genetic axis-epigenetic landscape (Fig. 1, 6, S14)—The authors wisely removed the evolutionary branching schematics in these cartoon figures. Now, more attention must be paid to the illustration of their conceptual paradigm. Fig. 1 schematizes the genetic axis as a linear path, one assumed to reflect the chronological accumulation of mutations. However, the vertical axis of Fig. 6 does not appear to encode any such phylogeny and instead is used a simple means to spread out the PC9 derivatives that were studied. Likewise, for the epigenetic landscape, the authors make a big deal about transitioning to adjacent basins of attraction, but what does left-vs.-right on the axis imply? Could all the cartoons be flipped or translated horizontally without any loss of meaning? Are the red-green-blue basins in Fig. 1 supposed to line up vertically or are they NOT supposed to line up? In Fig. 6, what evidence is there that DS3 is state that mutates into DS8? In Fig. S14, the implication is that DS8 derives from DS9. If the reader is not supposed to read into distance or location on the horizontal or vertical axes, what is the point of having the different PC9 derivatives share common axes at all?

<u>Response</u>: We thank the Reviewer for giving us the opportunity to clarify these important issues. The Reviewer is correct that a chronological accumulation of mutations is implied along the genetic axes in FIGS. 1 and 6, although the chronology need not be linear, despite how it is depicted in FIG. 1. In the previous versions of the manuscript, to avoid implying a linear chronology of mutations in our PC9 model system, we spread the genetic states out on the vertical axis of FIG. 6, as the Reviewer notes. We recognize now, however, the potential for confusion in displaying the states in this way, given our illustration in FIG. 1. Thus, to rectify this, we have modified FIG. 6 in the revised manuscript by adding a cartoon phylogenetic tree on the genetic axis to illustrate relative distance and separation of the different genetic states in the PC9 family. Specifically, we have included a common PC9 founder population (grey), from which both PC9-VU (blue) and PC9-MGH (light green) arise. We have placed PC9-VU closer to the founder in the tree than PC9-MGH based on the results of our genomic analyses (see response to Major Point #2 below). We also include PC9-BR1 (red) and DS8 (dark green) as parallel branches off of PC9-VU, with PC9-BR1 further from PC9-VU since we know it was derived and isolated many years ago while DS8 presumably emerged only recently. We have updated the caption to Fig. 6 to convey these points and have also modified the caption to FIG. 1 to clarify that states along the genetic axis do not generally align linearly.

For the epigenetic landscapes, the x-axis (labeled "molecular state" in the figures) represents position within a high-dimensional molecular state-space on which the quasi-potential energy of the system is defined. In potential energy diagrams common in physical chemistry, chemical

reactions are often depicted as reactant and product basins separated horizontally by an intervening potential energy barrier. The x-axis in these diagrams is usually referred to as the "reaction coordinate" to convey that a path (not necessarily linear) exists connecting these two states within the state-space. We are simply illustrating epigenetic landscapes in an analogous manner, which is common (e.g., PMID 23640024). Therefore, while in our cartoon representations in FIGS. 1 and 6 and Supplementary FIG. S13, flipping or horizontally translating the landscapes would not change the basic message of the illustrations, position on the x-axis does have a well-defined meaning within the framework of epigenetic landscapes and rotations or translations of the landscapes cannot, in general, be performed without changing the definition of the x-coordinate. We touch upon the issue of biochemical reaction networks, molecular states, and their relationships to the epigenetic landscape in the second paragraph of the Introduction and in somewhat more detail in the Supplementary Text (see "Relationship between transcriptomics and epigenetics"). For the sake of brevity, in previous versions of the manuscript we purposely limited these discussions and supplemented them with numerous references to published works on the subject. We retain this in the revised manuscript but have modified the caption to FIG. 1 to state explicitly that the x-axis of the epigenetic landscape represents position within a high-dimensional molecular state-space defining the quasi-potential energy.

Regarding the relative positions of the epigenetic basins in FIG. 1 across the different genetic states, we created the illustrations to explicitly show the basins *not* in alignment. We failed to mention this in the figure caption, however, for which we apologize. We have modified the caption in the revised manuscript to rectify this. Briefly, our reason for not aligning the basins in FIG. 1 is that, as discussed in the manuscript, genetic mutations alter the topography of the epigenetic landscape, which can include increasing the depths or heights of basins and barriers, the locations of basins, or even the number of basins. Thus, there is no *a priori* way of knowing that the basin locations will remain the same after a mutational event and, in fact, one would expect them to change, at least slightly. We actually believe this is an important and often overlooked fact that should be accounted for when comparing, e.g., molecular subtypes of the same tumor type across different cell lines and patient samples, which will invariably have somewhat different genetic backgrounds. This is relevant to ongoing work in our lab involving another cancer type.

Finally, we apologize for giving the impression in FIG. 6 that the mutation that led to DS8 came specifically from DS3. That was not our intention, we simply meant to draw an arrow from the PC9-VU epigenetic landscape to the DS8 genetic state to represent the mutational event within PC9-VU that led to DS8. To clarify this, we have modified FIG. 6 in the revised manuscript to show three dashed lines, one from each basin in the PC9-VU epigenetic landscape, converging at the base of an arrow pointing to the DS8 genetic state. Each dashed line is annotated with a question mark to signify that we do not know from which basin the DS8 mutation originated. Please note that we have not drawn the arrow directly from the DS7/DS9 basin because, as discussed in the Supplementary Text, we do not currently have enough evidence to make any definitive claim as to the specific origin of the DS8 state. We are simply arguing in the main text, based on the presented genomic, transcriptomic, and drug-response data, that DS8 emerged from PC9-VU as a new genetic state at some point in the past.

We hope these responses and the modifications we have made to FIG. 6 and the captions to FIGS. 1 and 6 are sufficient to alleviate the Reviewer's concerns regarding these matters.

2. Phylogenetic relationship between PC9-VU and PC9-MGH (Fig. S2B, S4C, S15)—The revised text gives the impression that PC9-VU and PC9-MGH had diverged from a common ancestor some unknown time ago (Fig. S2B). Lost in the weeds of the first submission was that "99% of the PC9-VU mutations were also seen in PC9-MGH" (Fig. S4C). Assuming the same genetic drift, does that not imply that PC9-VU is much closer to the ancestral line? Also, the main text should make clear that the identity of all the lines/clones was confirmed by the ~1e5 SNPs shared among PC9-MGH, PC9-VU, and PC9-BR1 (Fig. S4C); based on the language, the reader defaults to thinking that one of the lines has been misidentified. Last, it is unclear why the authors used SNPs (instead of somatic mutations not appearing as common variants in dbSNP) to evaluate the phylogenies in Fig. S15. Should not the PC9-BR1 and DS clones all derive from PC9-VU if these analyses are accurate?

<u>Response</u>: We agree with the Reviewer that the significant overlap between the PC9-VU and PC9-MGH mutations suggests that PC9-VU is closer to the PC9 founder line than PC9-MGH. As mentioned in our response to Major Point #1 above, we have modified the cartoon illustration in FIG. 6 to convey this point. Furthermore, per the Reviewer's suggestion, we have added language to the main text (2nd paragraph, 2nd subsection of Results) to explicitly state that the majority of SNPs are shared among the three cell line versions and, hence, confirms that they are related through a common founder population. We hope this alleviates any concerns regarding misidentification of cell line versions.

With regard to the phylogenetic analysis in Supplementary FIG. S13 (of the revised manuscript), we understand the Reviewer's concern that one would expect to see PC9-VU and PC9-MGH as separate branches in the phylogenetic tree and PC9-BR1 and the DS clones deriving from the PC9-VU branch. We suspect the reason why we do not see this is because of differences in the accuracy of variant detection between the heterogeneous PC9-VU and PC9-MGH parental populations and the more homogeneous DS clones. Since the parental lines are collections of clones, mutations unique to each clone are more diffuse within the parental populations and, hence, may only be detected at low confidence or not at all. This would amplify differences between the parental populations and the DS clones in the clustering analysis, as we see. We suspect PC9-BR1 clusters with the clones rather than the other parental lines because of its much larger mutational burden relative to PC9-VU and PC9-MGH (FIG. 3B,C of the main text), partially counteracting the effect of heterogeneity. However, despite these limitations of the analysis, we are still able to draw the useful insights that (i) PC9-VU and PC9-MGH are much more genomically similar to each other than they are to PC9-BR1, (ii) DS8 is genomically distinct from the other parental lines and the DS clones, and (iii) the DS clones are all very genomically similar to each other. These insights support the main conclusions of our manuscript, which is why we have included these results in the supplementary material.

To address the Reviewer's question directly regarding our use of SNPs, we used bi-allelic SNPs because they are high-confidence variants and are the default input data type for the tool that we used (SNPRelate). However, to assess whether including additional mutation classes would alter the principle component analysis or the phylogenetic tree structure shown in Supplementary FIG. S13, we reran the analysis using the tool in an alternate mode (copy.num.of.ref) that included not just bi-allelic SNPs but also lower-confidence variants, i.e., multi-allelic SNPs, indels, and structural variants. We also eliminated the trimming steps that remove autosomal and monomorphic SNPs.

These modifications had minimal effect on the results of the analysis (compare figures below to Supplementary Fig. S13).

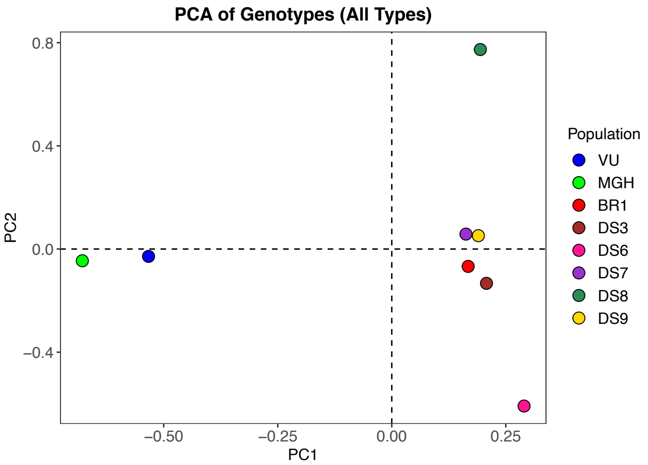

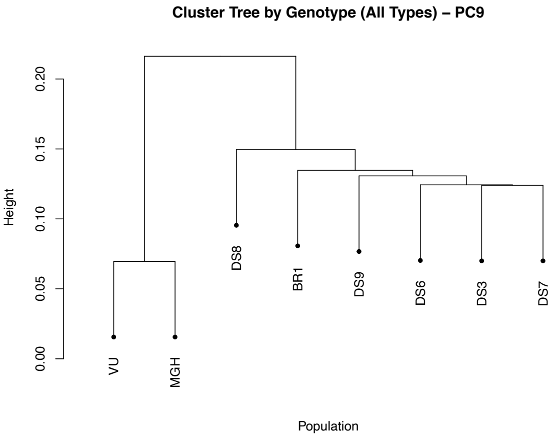

3. Inappropriate use of inferCNV (Fig. 3E, 4E)—This reviewer appreciates the use of inferCNV to estimate copy number, but it is important to remind that it is only an estimate that depends on how the algorithm was deployed. Inferring gains-losses of the DS clones by using PC9-VU as the reference (Fig. 4E) is acceptable for estimating chromosomal changes relative to the parental population. Doing the same for PC9-MGH, PC9-VU, and PC9-BR1 using their average gene expression is just wrong, because all it does is map gene expression differences among the three lines to their position on chromosomes. Estimation of true gains-losses would require some type of normal reference, which would be difficult for NSCLC. A better substitute in this circumstance would be to estimate local copy number profiles directly from the copy-number estimates of the bulk whole-exome sequencing for the three.

<u>Response</u>: We thank the Reviewer for raising this important point regarding CNV detection. As noted, we do not have a normal reference for the PC9 cell line versions because we do not have a sample of a common ancestor. While not ideal, in cases like this the developers of inferCNV do suggest using the average expression across cells as a baseline. The following quote is taken from their project GitHub Wiki ([http://github.com/broadinstitute/ inferCNV/wiki/Running-InferCNV](http://github.com/broadinstitute/inferCNV/wiki/Running-InferCNV)):

"Note, if you do not have reference cells, you can set ref_group_names=NULL, in which case the average signal across all cells will be used to define the baseline. This can work well when there are sufficient differences among the cells included (i.e., they do not all show a chromosomal deletion at the same place)."

It is for this reason that we performed the analysis on the cell line versions as we did. Because we were able to clearly identify amplifications and deletions across different chromosomal regions in PC9-MGH (which shares many mutations with PC9-VU), we deemed the cell populations to have the "sufficient differences", referenced in the comment, necessary for the analysis to be valid. We have modified the text in the revised manuscript (4th paragraph, 2nd subsection of Results), to clearly state that since a natural reference for the cell line versions does not exist, we have used average expression as the baseline based on the recommendation of the tool developers. We have

also included the qualifier "Within these analytical limits, …" in the following sentence to convey that this was not the ideal way to run the analysis and have updated the captions to FIGS. 3E and 4E to state what the baseline references were in each case.

Furthermore, based on the Reviewer's comment regarding the use of whole-exome data rather than scRNA-seq data for identifying CNVs, we performed an additional analysis using CoNIFER (Copy Number Inference from Exome Reads; PMID: 22585873). Using this tool, we identified 19 instances of CNVs across the PC9 cell line family members (see 1st table below). Most of these are in PC9-BR1 (7) and DS8 (8) and are in substantial agreement with the CNVs identified using inferCNV on the scRNA-seq data (compare 1st and 2nd tables below). In total, there are 11 shared mutations between the exome (CoNIFER) and scRNA-seq (inferCNV) derived CNVs (highlighted in grey in the tables below). Note that no instances of CNVs were identified in the exome data for PC9-MGH, although several were inferred from the scRNA-seq data (2nd table below). No CNVs were detected in PC9-VU using either approach.

CoNIFER – Exome (CNVs shared between two modalities are highlighted in grey)

	PC9-BR1	DS8	DS6	DS7
**# of CNVs**	7	8	3	1
**Loc-Type**	Chr2-del<br>Chr4-dup<br>Chr7-dup<br>Chr7-del<br>Chr8-dup<br>Chr10-dup<br>Chr17-del	Chr1-del<br>Chr6-dup<br>Chr8-dup<br>Chr10-dup<br>Chr11-del<br>Chr14-del<br>Chr17-dup<br>Chr19-dup	Chr2-dup<br>Chr4-del<br>Chr14-dup	Chr15-dup

inferCNV – Single-cell RNA-seq (CNVs shared between two modalities are highlighted in grey)

	PC9-BR1	PC9-MGH	DS8	DS6	DS3
**# of CNVs**	9	7	11	4	2
**Loc-Type**	Chr2-del<br>Chr3-dup<br>Chr4-dup<br>Chr7-dup<br>Chr8-dup<br>Chr9-del<br>Chr14-dup<br>Chr17-del<br>Chr22-del	Chr1-dup<br>Chr6-dup<br>Chr11-dup<br>Chr16-del<br>Chr19-del<br>Chr20-del<br>Chr22-dup	Chr1-del<br>Chr4-dup<br>Chr5-dup<br>Chr6-dup<br>Chr7-del<br>Chr8-dup<br>Chr10-dup<br>Chr14-del<br>Chr17-dup<br>Chr22-del<br>Chr22-dup	Chr3-del<br>Chr4-del<br>Chr15-del<br>Chr20-del	Chr9-del<br>Chr21-dup

On the whole, the results of our CoNIFER analysis (1st table above) using the exome data are generally consistent with the inferCNV results (2nd table above) and the rest of the genomic data

presented in the manuscript (FIGS. 3A–E and 4A–E): DS8 and PC9-BR1 are clearly genetically distinct from each other and the other parental lines (PC9-VU and PC9-MGH) and some sublines have a small number of variants that do not appear to have an impact on phenotype. We cannot currently explain why PC9-MGH shows instances of CNVs in the scRNA-seq data and not in the exome data but we note that the field of CNV detection is relatively new and the methods are not very sensitive nor mature. Whatever the case, the larger point we make in the manuscript is that the genomic data indicate that the cell line versions and DS8 are genetically distinct while the other sublines are not. We do not believe that the discrepancy reported here between CoNIFER and inferCNV detracts from this main point nor does this point hinge on the results of the CNV analysis. Thus, we trust that by performing this additional analysis, using a different tool and data type, and showing that we get qualitatively the same results, we have sufficiently allayed any concerns regarding the validity of the CNV results presented in the manuscript. Please note that for the sake of brevity and because we do not see the CoNIFER analysis as materially changing the message of our manuscript, we have not included the above results in the revised manuscript. We have provided them here, however, along with a detailed description below of how we performed the analysis, for the benefit of the Reviewer.**

<u>Methods: Whole Exome Copy Number Variant (CNV) Analysis</u>

Aligned, sorted, and indexed BAM files (intermediate output files from the WES sequence variant analysis) were generated and analyzed using CoNIFER (PMID: 22585873). A probes file (start and stop locations on each chromosome for each gene) was downloaded from the CoNIFER tool website (http://conifer.sourceforge.net/tutorial.html). BAM and probes files were input into the rpkm command to create relative exon abundances (RPKM files) at probe gene locations for each sample. Sample RPKM files were input into the analyze (svd=2) command to output a single HDF5 analysis file which compiles the Z- and SVD-transformed RPKM values for each exon in all samples. The analysis file was input into the call command to identify CNVs based on a tool-specific detection algorithm (~1 SVD-ZRPKM over average). Calls were visualized using the plot command, which highlighted regions around detected CNVs colored by sample name. A visualization for an example DS8 duplication is shown below.

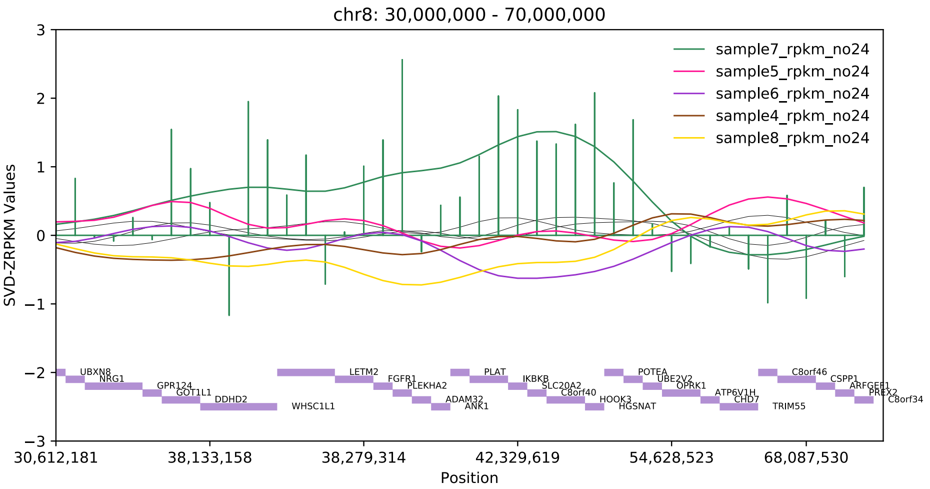

4. Inappropriate use of UMAP (Fig. S7C, S7F)—UMAP is a fine way to reduce dimensions to preserve local and global relationships, but the dimensions should not be used to calculate subtle distances between centroids (the UMAP developer says as much: github.com/lmcinnes/umap/issues/92). These calculations should be removed.

Response: We agree with the Reviewer and have removed the centroid-centroid distance plots from Supplementary FIG. S6 (previously S7) of the revised manuscript. We have also modified the language in the Results section of the main text to state that the distances between single-cell clusters in FIGS. 3F and 4F are qualitative rather than direct measurements.

5. Overinterpretation of the stochastic birth-death simulations (Fig. 5, S13)—The choice of bootstrapped Anderson-Darling recommended by Reviewer #2 is fine as a metric to compare data and stochastic simulations. However, the language of the main text is backwards that of standard hypothesis testing for frequentist statistics. A p value greater than 0.05 does not provide "strong evidence that these sublines […] are monoclonal"; only a small p value could provide evidence that the sublines are NOT monoclonal. Instead, the results suggest that the data are consistent with monoclonal population, and Occam's razor argues against invoking more complicated population structures. Compared to the results text, the methods text does a better job describing what these simulations do and do not support.

Response: We agree and thank the Reviewer for pointing this out. To rectify this issue, we have changed the language in the main text (1st paragraph, last subsection of Results) from "…is strong evidence" to "…is consistent with the view" that the sublines are monoclonal.

Minor points

1. Although the manuscript is much more streamlined, there remain instances of superfluous information that is unhelpful. For example, how does Fig. S1 enhance our appreciation that some genomic differences matter and others do not? Elsewhere, Fig. S12 classifies a UMAP plot based on inferred cell-cycle phase. This analysis can be done, but how does provide "further evidence that DS3, DS6, and DS7/9 correspond to three distinct cell states"? What result from the cell-cycle categorization would have falsified that there are three distinct cell states?

Response: We agree with the Reviewer that Supplementary FIG. S1 is no longer necessary and have removed it from the revised manuscript. Our goal with this figure was to illustrate the difference between genomes that differ genomically (any nucleotide difference) and those that differ genetically (differences only in "impactful" genes that affect the phenotype of interest). However, we believe this distinction has been sufficiently conveyed in the main and supplementary texts, which have been improved since the original submission thanks to input from the three Reviewers. We also agree that the cell cycle phase analysis of Supplementary FIG. S12 is not necessary and have removed it as well. We included it originally because an interesting observation is that there appear to be three independent sets of cycling cells (three G1 groups, three G2/M groups, and three S groups) within the PC9-VU cluster, which is consistent with the notion that PC9-VU is a heterogenous population of subclones. However, we believe this point has already been sufficiently made with the other data presented.

2. There remains some confusing duplication in the revision. In the first glance of Fig. 3D, for instance, the question arises why so many gene rows appear to have no mutations. One presumes that it is the same gene rows as shown in Fig. 4D, but the figure captions do not clarify this point. Also, although the figure is too small for row labels, there could be a handful of spaced row numbers that could be used to interpret the figure alongside the row numbers of Table S2.

Response: We thank the Reviewer for the comment and the suggestion. Yes, it is correct that the rows in FIGS. 3D and 4D of the main text are the same and correspond to the gene list in Supplementary Table S2. We have modified the figure captions to make this clear. Also, as suggested, we have added numbers next to every 10th row in the heatmaps to assist with cross-referencing against the gene names in Supplementary Table S2.

Reviewer #2:
[identifies himself as Aaron S Meyer]

Hayford et al have substantially improved their manuscript by streamlining the text and figures. I appreciate the analysis provided to address my and the other reviewers' concerns. I have a couple of addressable concerns below and am supportive of publication after these minor adjustments.

Line 101: "…emerged in the absence of selective pressures." This statement could be a bit more exact. While no drug selection was applied, the cell culturing process itself or features of that environment could be providing selective pressures for continued cell line adaptation.

Response: We thank Dr. Meyer for this clarifying comment. We agree that selective pressures associated with cell culturing or the environment may have affected the cell lines in ways we are unaware of. Therefore, we have modified the cited text to read instead "…emerged in the absence of drug treatment".

Lines 324-328: Since the semantic similarity relies on input about the difference between sublines, is it not differently powered to find links when there is a greater or lesser difference? Perhaps it would be better to say there is a strong genomic-transcriptomic link in DS8, but then other lines either have a weak link or insufficient changes to establish a link?

Response: We thank Dr. Meyer for the comment and the suggestion. We agree that it is advisable to soften our language regarding the results of the semantic similarity analysis. Thus, we have changed the text in the lines referenced to the following: "*Taken together, these results suggest a strong connection between genomics and transcriptomics in DS8 but weaker or non-existent connections in the other sublines.*"

Reviewer #3:

The authors have addressed the majority of my concerns. The presentation of the results is now clearer and the manuscript is more concise, therefore I recommend its publication in PloS Biology.

Response: We thank the Reviewer for their insights and suggestions during the review process and for recommending our manuscript for publication.
